# Low dose radiation risks for women surviving the a-bombs in Japan: generalized additive model

**DOI:** 10.1186/s12940-016-0191-3

**Published:** 2016-11-24

**Authors:** Greg Dropkin

**Affiliations:** Independent researcher, Liverpool, England

**Keywords:** Low dose radiation, Cancer, Women, Thyroid, Uterus, A-bomb, GAM

## Abstract

**Background:**

Analyses of cancer mortality and incidence in Japanese A-bomb survivors have been used to estimate radiation risks, which are generally higher for women. Relative Risk (RR) is usually modelled as a linear function of dose. Extrapolation from data including high doses predicts small risks at low doses. Generalized Additive Models (GAMs) are flexible methods for modelling non-linear behaviour.

**Methods:**

GAMs are applied to cancer incidence in female low dose subcohorts, using anonymous public data for the 1958 – 1998 Life Span Study, to test for linearity, explore interactions, adjust for the skewed dose distribution, examine significance below 100 mGy, and estimate risks at 10 mGy.

**Results:**

For all solid cancer incidence, RR estimated from 0 – 100 mGy and 0 – 20 mGy subcohorts is significantly raised. The response tapers above 150 mGy. At low doses, RR increases with age-at-exposure and decreases with time-since-exposure, the preferred covariate. Using the empirical cumulative distribution of dose improves model fit, and capacity to detect non-linear responses. RR is elevated over wide ranges of covariate values. Results are stable under simulation, or when removing exceptional data cells, or adjusting neutron RBE. Estimates of Excess RR at 10 mGy using the cumulative dose distribution are 10 – 45 times higher than extrapolations from a linear model fitted to the full cohort. Below 100 mGy, quasipoisson models find significant effects for all solid, squamous, uterus, corpus, and thyroid cancers, and for respiratory cancers when age-at-exposure > 35 yrs. Results for the thyroid are compatible with studies of children treated for *tinea capitis*, and Chernobyl survivors. Results for the uterus are compatible with studies of UK nuclear workers and the Techa River cohort.

**Conclusion:**

Non-linear models find large, significant cancer risks for Japanese women exposed to low dose radiation from the atomic bombings. The risks should be reflected in protection standards.

**Electronic supplementary material:**

The online version of this article (doi:10.1186/s12940-016-0191-3) contains supplementary material, which is available to authorized users.

## Background

Analyses of cancer mortality and incidence in Japanese A-bomb survivors continue to play a key role in establishing radiation risk estimates. The 1958–1998 incidence cohort was analysed by Preston and co-workers [1], whose main model assumes a linear dose response. Their study found some evidence of radiation risk at doses below 150 mGy, and higher risks for women. Previously Pierce and Preston [2] found a significant dose response for all solid cancer incidence in the 0 – 100 mGy range. However, according to the EPA Blue Book [3] “it has not been possible to demonstrate and quantify risk in the Lifespan Study (of A-bomb survivors) at doses below about 100 mGy”. The ICRP [4] recommended annual dose limit for occupational exposures is 20 mSv (whole body dose).

Some cancer studies find non-linear responses to radiation [5–8] and there are some significant results at low doses [9–16]. Low dose radiation risk was reviewed in 2003 [17] and remains a focus of research [18].

I consider cancer incidence in female Japanese A-bomb survivors in the dose ranges 0 – 500 mGy, 0 – 100 mGy, and 0 – 20 mGy, using the grouped anonymous (public) incidence data for the 1958 – 1998 cohort.

Attained age is the single most powerful predictor of cancer incidence. Models can be refined using birth cohort, age-at-exposure or time-since-exposure, city, urban/rural residence, and dose. The Not In City (NIC) group of Hiroshima and Nagasaki residents who were out of the area at the time of bombing can be included.

The analysis uses the Generalized Additive Model [19] implemented in R [20] through the programme mgcv [21]. GAM is a flexible technique for fitting non-linear responses and covariate interactions. Models are investigated for all solid cancers, and then applied to the most common anatomical or histological groups and specific sites within the female incidence data: digestive, genital, respiratory, adenocarcinoma, other epithelial, squamous, stomach, colon, liver, pancreas, gallbladder, rectum, uterine, cervical, uterine corpus, ovarian, lung, breast, thyroid, and central nervous system.

The analysis aims to test for linearity of the dose response, explore the interaction of dose with attained age, age-at-exposure and time-since-exposure, adjust for the highly skewed distribution of dose, test whether the response is significant below 100 mGy, and estimate the risks at 10 mGy.

## Methods

The public dataset *LSSinc07* [22] was downloaded from the Radiation Effects Research Foundation (RERF) [23]. Records with unknown dose were excluded, leaving 25,570 cells with grouped data on 17,448 solid cancers among 105,427 survivors with 2,764,735 person-years of follow-up. The covariate **py** denotes person-years observation in each cell. **logage** is the log of **age**, the attained-age covariate which RERF obtained by weighting each individual contribution by its p-y within the cell. Likewise **agex** is the p-y weighted mean age-at-exposure, **since** is the difference between **year**, the p-y weighted mean calendar time, and the exposure date (depending on city), and **birth** is defined as year - age. The dataset also contains sex, city, and distcat, the latter taking the values 1 (urban), 2 (rural), and 3 (Not in City). The grouped data is stratified by these (and other) categories. The dataset is then restricted by sex = 2, and by distcat < 3 if the NIC group is excluded; city and distcat are converted to factor variables **fc** and **fd**. For convenience, covariate names are introduced in bold, but then used in normal font.

When modelling all solid cancers, the instantaneous flash dose is the p-y weighted mean colon dose in mGy, defined as **coldose** = 1000*cola02w10 using the DS02 dosimetry and a Relative Biological Effectiveness (for neutrons) of 10, unless otherwise specified. The dataset is then restricted by coldose < 100 (or < 20 or < 500). For convenience A^+-^, B^+-^ and C^+-^ refer to 0 – 20 mGy, 0 – 100 mGy and 0 – 500 mGy subcohorts including or excluding NIC. Weighting each coldose by the py of its data cell, the mean coldose in A^+-^, B^+-^ and C^+-^ is 2.43, 3.70, 9.84, 13.85, 36.44, 48.93 mGy respectively.

More generally, modelling uses the appropriate organ dose as in [1] and *LSSinc07*. Tissue weighting factors are not applied. When considering a cancer group or site, **dose** refers to the relevant p-y weighted organ dose in mGy, and the response variable **obs** is the number of incident cases of that cancer in each data cell. The case numbers by site and dose range are shown in Table 1. Note that A^+-^ etc. are defined from coldose, no matter which cancer site is under consideration.

**Table 1:** Case numbers by site and dose range

Site	A^-^	A^+^	B^-^	B^+^	C^-^	C^+^	Mid age	Mid agex	Mid since
Solid	4239	6271	5474	7506	6712	8744	68.4	33.1	36.9
Adeno	2477	3722	3188	4433	3928	5173	67.0	28.3	38.6
Othepi	1016	1460	1310	1754	1583	2027	73.3	38.4	32.7
Squam	550	823	718	991	881	1154	63.5	33.2	28.6
Digest	2206	3263	2825	3882	3418	4475	71.7	33.5	38.6
Genital	660	1008	871	1219	1035	1383	61.8	31.6	28.6
Resp	324	487	441	604	558	721	73.2	36.5	38.6
Stom	997	1459	1285	1747	1564	2026	71.6	36.6	33.6
Colon	373	578	471	676	570	775	71.7	29.3	44.1
Liver	287	416	356	485	433	562	71.7	32.6	41.4
Gall	181	266	234	319	273	358	73.2	36.8	38.6
Panc	134	192	172	230	208	266	73.4	36.6	38.6
Rect	183	283	245	345	291	391	68.5	31.6	41.3
Uterus	519	806	689	976	821	1108	61.7	31.5	28.6
Cervix	384	598	509	723	603	817	61.8	31.7	28.5
Corpus	84	132	108	156	126	174	61.1	23.1	38.5
Ovary	115	170	143	198	171	226	63.5	31.6	33.6
Breast	441	667	554	780	713	939	59.1	22.4	38.6
Lung	290	437	396	543	503	650	73.3	36.4	38.6
Thyroid	158	219	203	264	271	332	66.7	28.2	31.9
Cns	93	117	125	149	151	175	66.2	26.7	37.0

Mid age (agex, since) is the age (agex, since) below which 50% of cases occur.

Values are shown for B^+^, and are similar for other dose ranges

Modelling seeks to predict the cancer risk, i.e. the expected value of obs/py, as a function of covariates and their interactions. Each data cell is regarded as an independent source of Poisson distributed incident cases, and penalised regression with cubic splines, using a log link, is used to estimate the expected value of obs. Briefly (see [21]):

obs_j_ ∼ Pois(*μ*_j_) where j indexes the data cells

log(*μ*_j_) = $\sum $_i_ *β*_i_f_i_(x_j_) where i indexes the model components f and coefficients *β*; f_i_ is a function of covariates x, whose form is specified in the model but whose value varies with x_j_ in cell j; and *β*_i_ is a numerical coefficient of f_i_, independent of j, to be estimated during model fitting.

The f_i_ can include offsets, whose *β*_i_ is fixed at 1; factors; parametric terms; splines derived from the covariates; and tensor products of such splines.

A cubic regression spline with components f_0_, f_1_…f_k-1_ is obtained from covariate x by placing k knots, for example at equally spaced quantiles of x, forming the natural cubic splines with value 1 at the specified knot and 0 at the other knots, using a centering constraint to achieve $\sum $_j_f_i_(x_j_) = 0 when i > 0, and setting f_0_ = 1.

During model fitting, the *β*_i_ are estimated by minimising the penalised deviance, Dev + *β* ^T^S *β*, where the positive semi-definite penalty matrix S is a direct sum of blocks generated from the splines, each block incorporating a smoothing parameter *λ*_b_. For tensor product splines, there are separate smoothing parameters for each margin. Dev is the Deviance, given in the Poisson case (omitting subscripts j) by

-2$\sum $[obs ·log(*μ*)- *μ* - I_obs_ ·(obs ·log(obs)-obs)] where I_obs_ = 1 if obs > 0 and 0 otherwise

Minimising the penalised deviance is a compromise between approximating the data (lowering Dev), and the “wiggliness” of the fitted model as measured by the penalty. Higher values of the smoothing parameter(s) impose stronger penalties, making the fitted model smoother with lower “effective degrees of freedom” (a penalised version of the number of independent parameters), but allowing it to stray further from the individual data points. As *λ*_b_ →*∞*, the model becomes log-linear (only the linear components of the spline terms are unpenalised by S).

Model fitting involves optimising the smoothing parameters affecting S, through a criterion such as Generalized Cross Validation, Restricted Maximum Likelihood, or Marginal Likelihood (see [21, 24] for details). After fitting, inference is based on the Bayesian posterior covariance of *β*.

With the fitted $\hat {\beta }_{i}$, the expected value of obs_j_ is *μ*_*j*_=$\exp (\sum $_i_ $\hat {\beta }$_i_f_i_(x_j_)). The fitted model also predicts the expected value *μ*_j,0_ if the dose covariate is reset to 0 while preserving all other covariates and the $\hat {\beta }$_i_. In each cell, baseline risk is estimated internally from the fitted model as *μ*_j,0_ / py_j_ and Relative Risk is RR_j_ = *μ*_j_ / *μ*_j,0_.

From here on, models are defined and fitted using the R programme mgcv. For example, the initial Poisson model is specified as

$${} \begin{array}{ll} P0 &<\!\!\text{-}\ gam\left(obs \sim offset(log(py))+(fc+fd)^{\wedge}2\right.\\ &\quad+ti(logage,k=10)+ti(birth,k=10)\\ &\quad+ti(logage,birth,k=10),\\ &\quad\left. family=\verb+"+poisson\verb+"+, method=\verb+"+ML\verb+"+\right.) \end{array}  $$

The reverse arrow <- defines the model by the subsequent expression, with gam denoting a generalized additive model. Here, obs is regarded as a sample from a Poisson distribution whose mean, varying across the data cells, is specified with a (default) log link. The offset term, with coefficient fixed at 1, ensures that the fitted value of obs/py is independent of py. The factor variables and their first order interaction are included as (fc+fd)ˆ2. The smooth term ti(logage) refers to an unknown linear combination of splines derived from logage, and the tensor product term ti(logage, birth) allows for interaction of logage with birth. In each smooth, the basis dimension is set at 10. By default, “cubic regression splines” are used with the ti terms. The smoothing parameter optimisation method indicated by ~ML~ is Marginal Likelihood. For details see [21] and ?gam and ?ti (within mgcv). Setting family = ~quasipoisson~ allows a mean-variance relation V= *μ* *ϕ* where V is the variance about mean *μ* and *ϕ*>0 is a scale factor. For the Poisson family, *ϕ* = 1, but any positive scale can be set, or estimated as below.

The dose covariate is highly skewed and, in the grouped data, multimodal. Over 70% of py in the full data has dose < 20 mGy, or over 60% if NIC cells are excluded. Likewise over 84% has dose < 100 mGy, or 79% if NIC cells are excluded. The grouped data cells are also stratified by dose category, an analytical decision embodied in the anonymous public data. For example, each cell in A^-^ or A^+^ will contain records with dose either below 5 mGy or between 5 and 20 mGy.

**Fig. 1:**
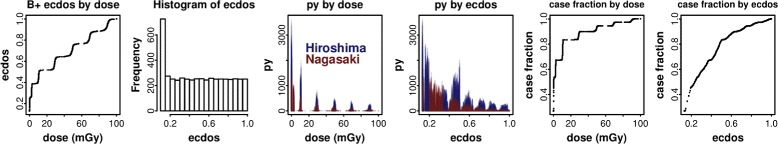
Empirical cumulative distribution of dose. The ecdf of dose, denoted ecdos, is plotted against dose in B^+^ (0 - 100 mGy including Not in City). The histogram is approximately uniform. py and case fractions are plotted against dose and ecdos

To adjust for this irregular distribution, when dose = D define **ecdos** as the proportion of cells with dose ≤ D, a monotone increasing function of dose with an approximately uniform distribution. It increases quickly where dose is concentrated, remaining static where dose is sparse. While max(**ecdos**)=1, min(**ecdos**) > 0 due to cells with dose = 0. Formally, **ecdos** is the empirical cumulative distribution function of dose, obtained in R as ecdf(dose)(dose), with inverse transformation quantile(dose,). Figure 1 shows **ecdos** and dose in B^+^.

The initial model P0 (and its quasipoisson variant Q0) are enlarged with terms using ecdos or dose and interactions, as shown in Table 2.

**Table 2:** Model definitions

Model	Family	*ϕ*	Model	Family	*ϕ*	Terms
P0	poisson	1	Q0	quasipoisson	>0	offset(log(py))+(fd+fc)ˆ2+ti(logage) +ti(birth)+ti(logage,birth)
P1e	poisson	1	Q1e	quasipoisson	>0	.+ecdos
P2e	poisson	1	Q2e	quasipoisson	>0	.+ti(ecdos)
P3e	poisson	1	Q3e	quasipoisson	>0	.+ti(ecdos)+ti(ecdos,logage)
P4ae	poisson	1	Q4ae	quasipoisson	>0	.+ti(ecdos)+ti(ecdos,agex)
P4se	poisson	1	Q4se	quasipoisson	>0	.+ti(ecdos)+ti(ecdos,since)
P5ae	poisson	1	Q5ae	quasipoisson	>0	.+ti(ecdos)+ti(ecdos,logage) +ti(agex)+ti(ecdos,agex)
P5se	poisson	1	Q5se	quasipoisson	>0	.+ti(ecdos)+ti(ecdos,logage) +ti(since)+ti(ecdos,since)
P1d	poisson	1	Q1d	quasipoisson	>0	.+dose
P2d	poisson	1	Q2d	quasipoisson	>0	.+ti(dose)
P3d	poisson	1	Q3d	quasipoisson	>0	.+ti(dose)+ti(dose,logage)
P4ad	poisson	1	Q4ad	quasipoisson	>0	.+ti(dose)+ti(dose,agex)
P4sd	poisson	1	Q4sd	quasipoisson	>0	.+ti(dose)+ti(dose,since)
P5ad	poisson	1	Q5ad	quasipoisson	>0	.+ti(dose)+ti(dose,logage) +ti(agex)+ti(dose,agex)
P5sd	poisson	1	Q5sd	quasipoisson	>0	.+ti(dose)+ti(dose,logage) +ti(since)+ti(dose,since)

Models use cubic regression (cr) splines, with basis dimension 10

Model fitting by method ML

“.+” : augment the initial model P0 (or Q0) by the terms which follow

ti refers to a smooth term on the enclosed covariate(s)

The nested chains P0 ⊂ P2e ⊂ P3e ⊂ P5ae, P0 ⊂ P2e ⊂ P4ae ⊂ P5ae, P0 ⊂ P2e ⊂ P3e ⊂ P5se, and P0 ⊂ P2e ⊂ P4se ⊂ P5se give models of increasing complexity using ecdos with agex or since respectively. Corresponding nested chains P0 ⊂ P2d etc. are defined using dose instead of ecdos. P2 refers to the pair P2e and P2d, etc.

The scale for a quasipoisson model M is estimated iteratively via Pearson $\chi ^{2} = \sum (obs-fit)^{2}/fit$, where fit is the fitted value, by seeking $\hat {\phi } \simeq \chi ^{2}/dfres$ where dfres is the residual degrees of freedom (# of records – effective degrees of freedom).

Additional file 1: Appendix A describes methods for Relative Risk RR, confidence intervals based on the Bayesian posterior distribution of *β*, dose response curves, and model selection. Simulation tests used to choose the preferred method of smoothing parameter optimisation and to validate the models are outlined in Additional file 1: Appendix B.

Numerical results are given at 2.5 and 10 mGy in A^+-^, 2.5, 10, 20, 40, 60, 80 mGy in B^+-^, and at 2.5, 10, 100, 200, 300, and 400 mGy in C^+-^. Confidence intervals are 90% (lower and upper 95% confidence limits LCL95% and UCL95%) unless otherwise specified, and “borderline” denotes 0.98 ≤ LCL95% ≤ 1.

Initially, no minimum value of the smoothing parameters was imposed. However, if the visual output was “too wiggly”, indicating overfitting, min.sp was increased until the output appeared fairly smooth, a subjective judgement.

In each data cell, a fitted model gives predicted values *μ*, with $\sum {\mu } = \sum {obs}$, and predicts values *μ*_0_ if dose had been reset to 0, or ecdos reset to ecdf(dose)(0). The attributable fraction $1 - \sum {\mu _{0}}/\sum {\mu }$ is the predicted number of additional cases above the baseline, as a proportion of those observed. $\sum {\mu }/\sum {\mu _{0}}$ estimates the Standardized Incidence Ratio from the fitted model.

The age distribution of cases varies between cancer sites. Define the “mid” age (and agex, since) for a site and dose range as that below which half the cases occur. Table 1 includes the mid values by sites in B^+^; values for other dose ranges are similar. Results are reported at the mid age rounded to the nearest 5 years, and where appropriate at the rounded mid agex or since.

For a fitted model M, set I_M_ = 1 on any data cell for which LCL95% > 1, and 0 otherwise. The proportion of obs (or py) for which I_M_ = 1 measures the extent to which RR > 1 is significant across the dose range. Since I_M_ = 0 if dose = 0, the proportions are obtained for D_0_, those cells with dose > 0, or for D_0.5_ (dose > 0.5 mGy). Corresponding proportions are obtained on S_0.5_ (since < 35 and dose > 0.5 mGy), or T_0.5_ (agex > 35 and dose > 0.5 mGy), or S_0.5_ ∩ T_0.5_. Likewise, the indicator J_M_ = 1 on any data cell for which UCL95% < 1, and 0 otherwise. The proportion of obs (or py) for which J_M_ = 1 measures the extent to which RR < 1 is significant across the dose range.

Selected models for all solid cancers were re-fitted after excluding exceptional data points as detected by Cook’s Distance, or with thin plate regression splines, or for urban residents, or with RBE as obtained in [25].

For a fitted model M, influence(M) is a vector of leverage at each cell, and plotting influence(M1)/influence(M2) against dose compares model sensitivities in different dose regions. The ability of ecdos and dose models to detect hypothetical dose-responses was compared by simulation (Appendix B). Further simulations considered the uncertainty in dose.

These intensive computations were applied only for all solid cancers. Elsewhere, the Bayesian posterior CIs (Appendix A) are reported without further validation. Some codes with commentary are shown as additional files.

## Results

**Fig. 2:**
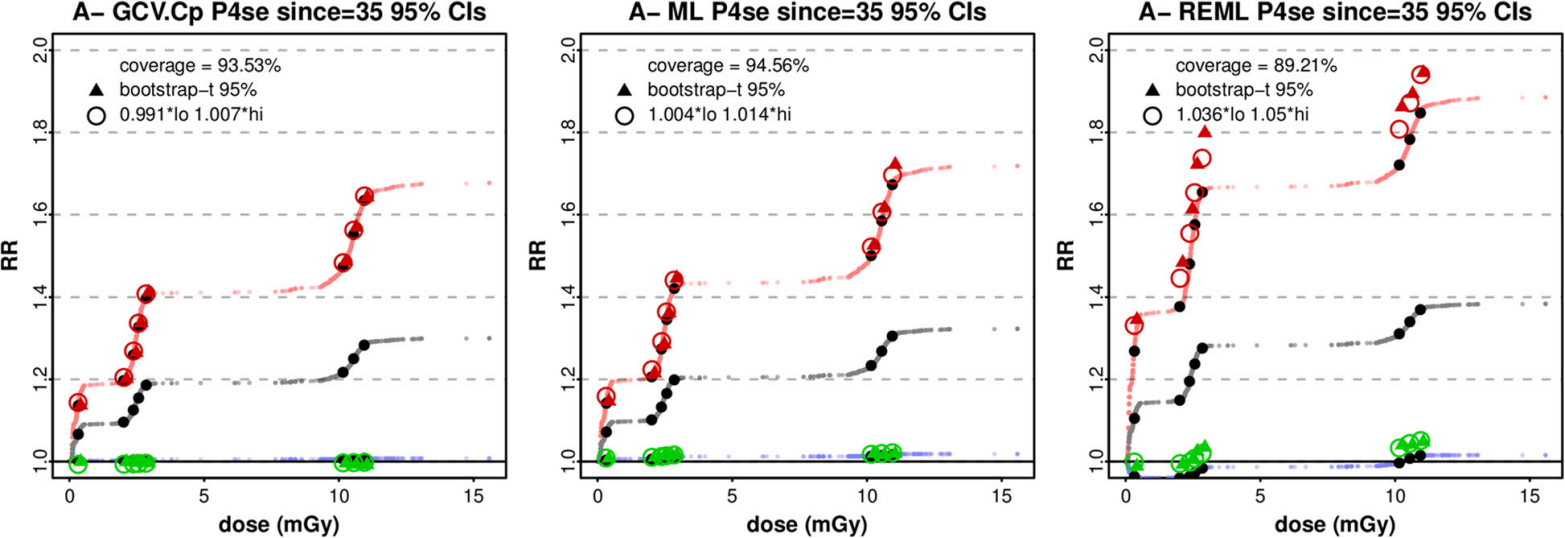
Optimisation methods and Bayesian posterior confidence intervals. For all solid cancers in A^-^ (0 - 20 mGy excluding NIC), the model P4se (see Table 2) is fitted with smoothing parameter optimisation by GCV.Cp, ML, and REML. At 35 years time-since-exposure, Relative Risk is shown by grey curves, and 95% Bayesian posterior confidence intervals (Appendix A) are shown by blue and red curves. Simulation gives estimates of coverage, Bootstrap-t CIs (triangles), and an alternative CI (large circles) stretched to achieve 95% coverage (Appendix B), with stretch factors as shown in the plot key

For all solid cancers, no minimum smoothing parameters were imposed. Basis dimension k=10 was sufficient when tested by gam.check or modelling residuals against covariates. The ML method of smoothing parameter optimisation was preferred to alternatives, giving 95% CIs which performed well under simulation tests described in Appendix B and shown in Fig. 2. ML is discussed in [24].

P5ae, P5se, P5ad and P5sd were cross-validated (Appendix B) in A^+-^, to test the proportion of deviance explained and the estimated RR |_10 mGy_ with 90% CIs, see Additional file 1: Table S1.

Additional file 1: Figure S1 shows the dose response in B^+^. All plots show RR significantly raised, although the response is lower with the dose models. Plots with REML or GCV.Cp optimisation methods are similar. For the ecdos models, the appearance of the response reflects the relation between ecdos and dose (see Fig. 1); corresponding curves of RR against ecdos are regular.

**Fig. 3:**
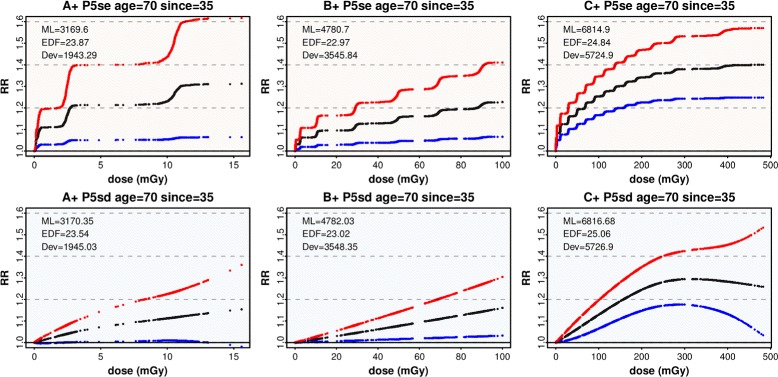
Dose response of preferred models in A^+^, B^+^, C^+^. For all solid cancers, the top row shows the Poisson model P5se selected in each dose range to minimise ML, with the corresponding dose model P5sd on the bottom row. RR and 90% CIs at the rounded mid since and age, are plotted against dose. ML scores, effective degrees of freedom, and Deviance are listed. For model definitions see Table 2

Figure 3 compares the responses of P5se and P5sd in A^+^, B^+^ and C^+^, evaluated at age 70 and since 35. Curves are similar over A^-^, B^-^ and C^-^, and for P5ae and P5ad at age 70 and agex 35, with slightly weakened significance.

Data simulated over B^-^ from a variety of hypothetical forms of the true dose-response curve, without interactions, was fitted with P2e and P2d (Appendix B). Table 3 has results for 10 curves, comparing models by the mean coverage of their respective CIs across the dose range. Models with ecdos give better or equivalent coverage, unless the true dose-response is linear.

**Table 3:** Coverage by P2e and P2d of true dose response

Name	ERR	*β*	*σ*	*τ*	*γ*	Form	Mean (lhe)	Mean (lhd)
N1	*g*(*d*,*β*,*σ*,*τ*)	0	0			null	95.18%	94.90%
L1	*g*(*d*,*β*,*σ*,*τ*)	0.002	0			linear	81.46%	94.56%
H1	*f*(*d*,*β*,*σ*,*τ*)	0.004	-0.2	0.05		hormesis	75.08%	69.86%
P1	*f*(*d*,*β*,*σ*,*τ*)	0	0.2	0.05		plateau	93.36%	69.68%
C1	*f*(*d*,*β*,*σ*,*τ*)	0.0012	0.08	0.5		two phase	90.88%	50.08%
C2	*f*(*d*,*β*,*σ*,*τ*)	0.015	0.75	0.2		two phase	94.80%	13.98%
R1	*g*(*d*,*β*,*σ*,*τ*)	0.002	0.01	0.08		two phase	93.64%	68.14%
R2	*g*(*d*,*β*,*σ*,*τ*)	0.002	0.01	0.04		two phase	93.98%	66.10%
T1	*h*(*d*,*γ*)				50	threshold	88.40%	87.66%
T2	*h*(*d*,*γ*)				25	threshold	86.88%	86.90%

All Solid cancers, fitting over B^-^

ERR: true value as specified by function

*f*(*d*,*β*,*σ*,*τ*)=*β*·*d*+*σ*·(1−*exp*(−*τ*·*d*))

*g*(*d*,*β*,*σ*,*τ*)=*β*·*d*+*σ*·*d*·*e* *x* *p*(−*τ*·*d*)

*h*(*d*,*γ*)=*ifelse*(*d*<*γ*,0,1/5·((*d*−*γ*)/(100−*γ*))^3^)

Mean (lhe or lhd): mean coverage at 10, 20…100 mGy of 95% CI for P2e (P2d) see also Appendix B

**Fig. 4:**
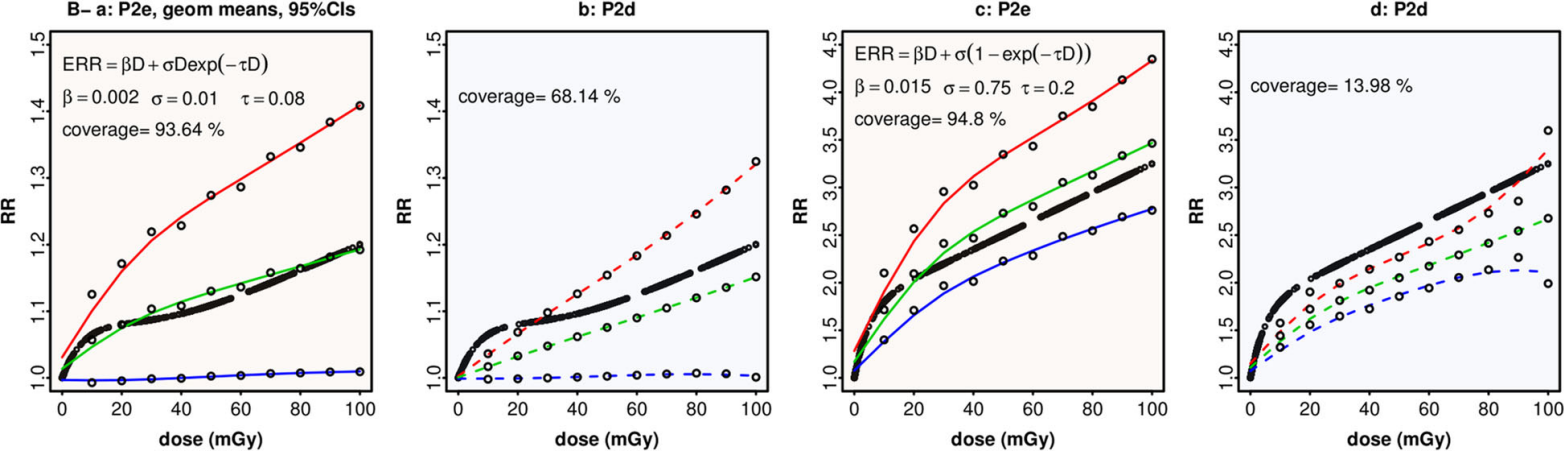
Ability to capture a known response. Data is simulated (Appendix B) over B^-^ with RR = 1 + ERR as specified by the formulas in Panels **a** and **c**, without interactions. Thick black curves show the true RR. Simulated data is modelled by P2e (Table 2) in Panels **a** and **c**, and remodelled by P2d (Table 2) in Panels **b** and **d**. At dose = 10, 20... 100 mGy, fitted values and 90% CIs are obtained at each simulation step. Coverage is the % over all 10 doses for which the simulated CI contains the true RR. Panels display the geometric means (*hollow dots*) interpolated by smoothing splines, *green* for fitted values, *blue* (*red*) for lower (upper) 95% CIs

Figure 4 displays the simulated geometric mean fitted values and CIs for two of these curves (R1 and C2), showing that the dose model underestimates the true response while the ecdos model captures it correctly.

The increased sensitivity of ecdos models at low doses is seen in Additional file 1: Figure S2, plotting the ratio of influence with P5sd to that with P5se, against dose. In B^+-^ and C^+-^ low doses have less influence on P5sd, reducing its sensitivity to their effects.

Splines constructed from ecdos have better variability than dose splines at very low doses, lowering the internal baseline estimates. Additional file 1: Figure S3 compares splines from P2d, P2e, and P2g constructed from gdos, a smooth approximation to ecdos, when modelling over B^+^. Although the P2g response curve is simpler, the parameters of gdos vary with dose range and its distribution is multimodal, whereas ecdos has a universal definition and an approximately uniform distribution.

**Fig. 5:**
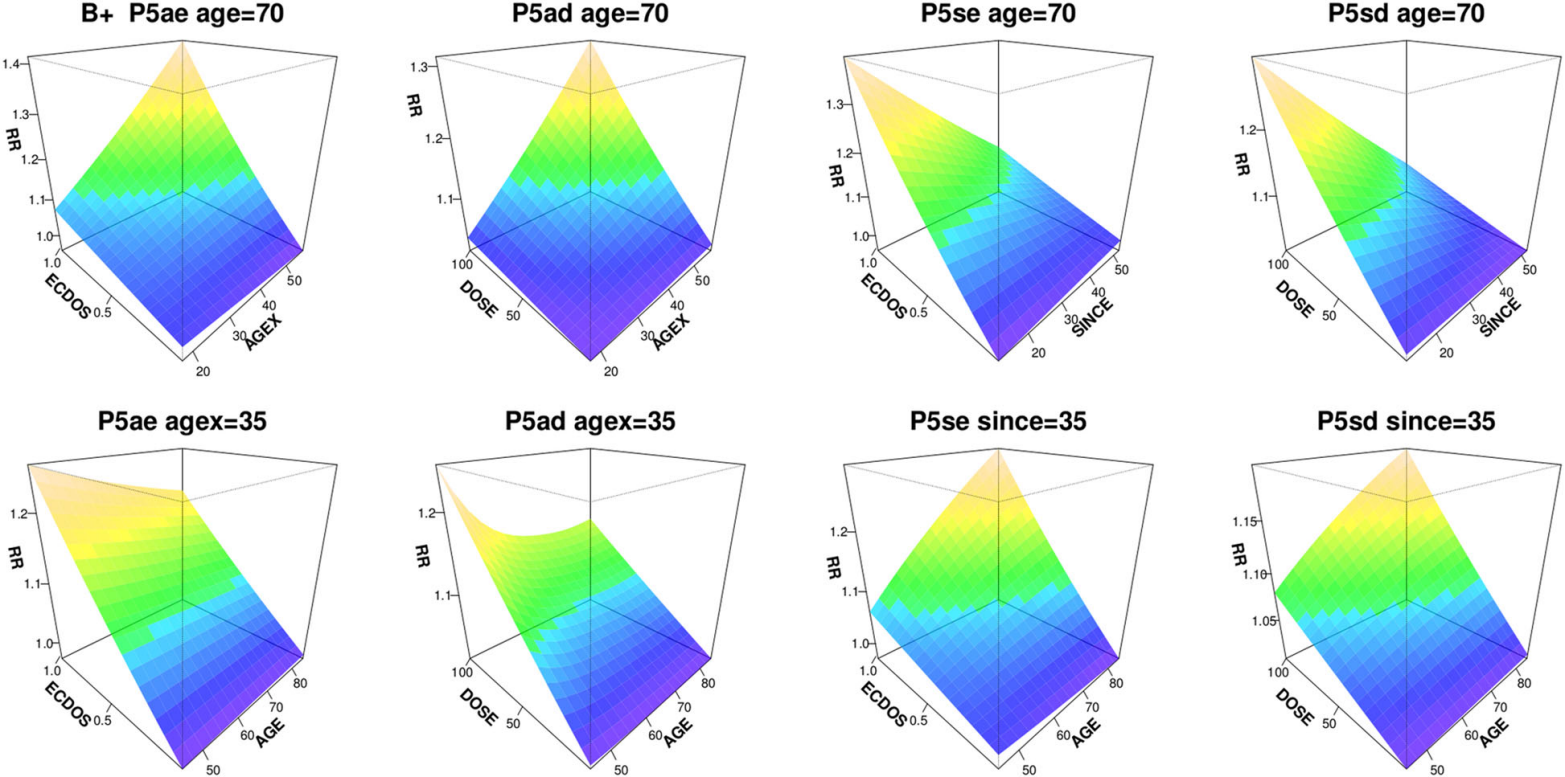
Interaction perspective plots in B^+^. For all solid cancers in B^+^, the ecdos and dose versions of P5a and P5s (Table 2) are fitted. The top row shows RR (z-axis) as a joint function of ecdos or dose (y-axis) and agex or since (x-axis), at age 70. Below, RR is shown as a function of ecdos or dose and age, at agex 35 or since 35. Shadings from purple to gold show increasing RR

**Fig. 6:**
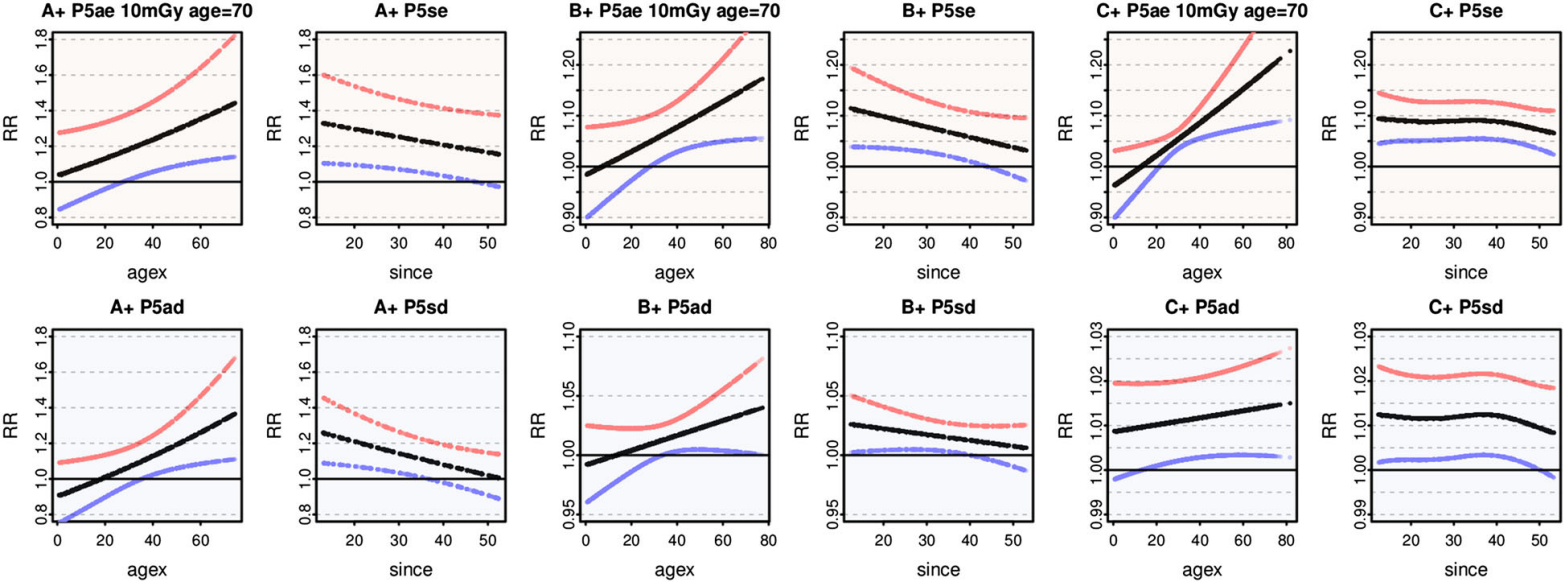
Interactions at 10 mGy. For all solid cancers in A^+^, B^+^, and C^+^, RR and 90% CI at 10 mGy is plotted against agex or since, at age 70, for the ecdos and dose versions of P5a and P5s (Table 2). The top row shows ecdos models, with corresponding dose models below

Interactions in B^+^ are shown by perspective plots in Fig. 5, fitting P5a and P5s with either ecdos or dose, and fixing age = 70 or agex = 35 or since = 35. Figure 6 shows RR |_10 mGy_ and 90% CIs at age 70, using P5a and P5s fitted over A^+^, B^+^ and C^+^. With P5a at age 70 RR increases with agex at all doses in A^+-^, B^+-^ or C^+-^. With P5s at age 70, RR decreases (or is constant) with since at all doses. The decrease is confined to since > 35 in C^+-^. With P5a at agex 35 RR generally decreases (or is constant) with age. With P5s at since 35 RR increases (or is constant) with age in A^+-^ and B^+-^, but decreases with age at higher doses in C^+-^.

Poisson model selection methods (Appendix A) were applied to choose preferred models. P5se is preferred in all six dose ranges by method 1 (lowest ML score). The three methods chose models with ecdos in 17 of 18 cases. Additional file 1: Table S2 shows the preferred Poisson models for each range and selection method. As appropriate, RR is shown at age 70, agex 35, or since 35. All preferred models show RR elevated at 2.5, 10, 20, 40, 60, 80, 100, 200, 300, and 400 mGy, with LCL95% > 1, except for method 2 in B^+^ where P3d is preferred, with LCL95% ≥ 1. In all cases Deviance < dfres. The proportion of Deviance explained varies from 46.1% in C^-^ to 68.1% in A^+^. The attributable fraction varies from 1.4% (method 2 in B^+^) to 9.4% in A^-^.

Additional file 1: Table S2 also shows the percentage of cases and py in D_0_ for which RR > 1, LCL95% > 1, or UCL95% < 1; likewise for cells in D_0.5_, S_0.5_, T_0.5_, or S_0.5_ ∩ T_0.5_ (see “Methods”). For each of these preferred models, cell and dose ranges, RR > 1 holds for over 80% of cases and py, and UCL95% < 1 never occurs. Except with method 2 in B^+^, LCL95% > 1 holds for over 70% of cases in T_0.5_, over 75% of cases in S_0.5_, and over 90% of cases in S_0.5_ ∩ T_0.5_.

**Fig. 7:**
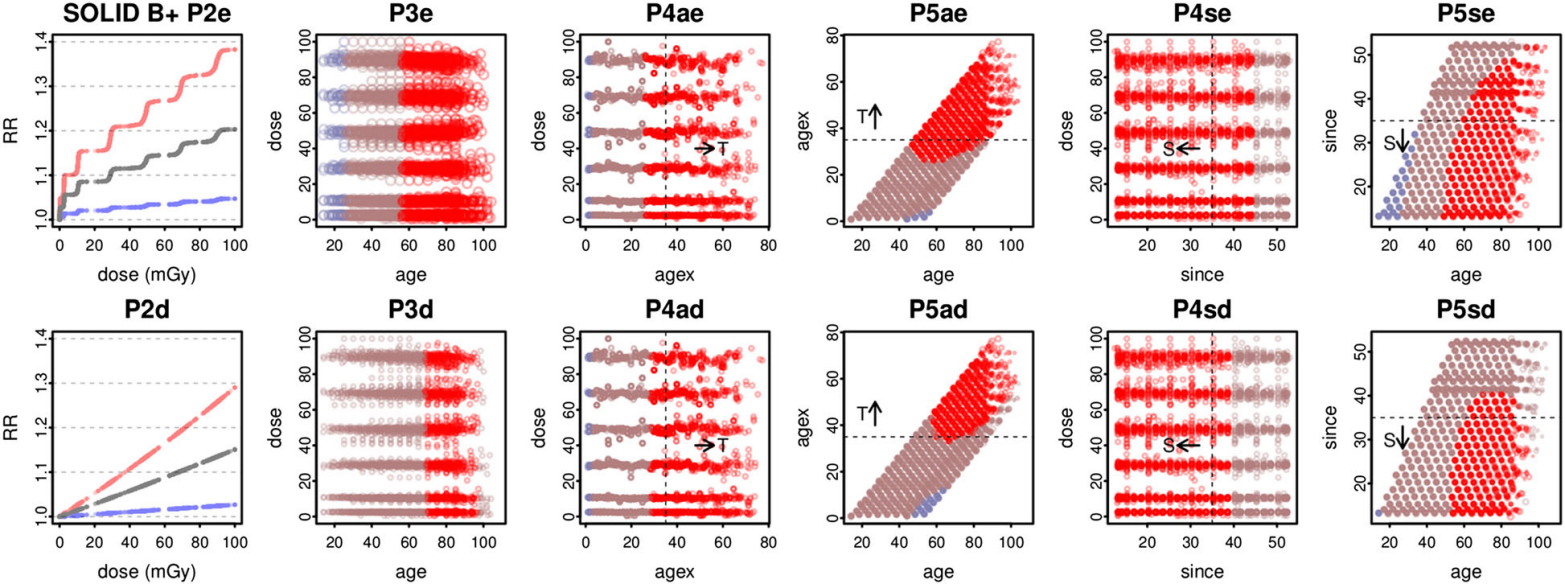
B^+^ significance regions. For all solid cancers in B^+^, Poisson ecdos and corresponding dose models (Table 2) are plotted on the top and bottom rows. RR and 90% CIs are shown for P2e and P2d. For the interaction models, covariate pairs are shaded red if the lower confidence limit LCL95% > 1, brown if RR > 1 but LCL95% ≤ 1, blue-grey if RR ≤ 1 but UCL95% ≥ 1, and bright blue if the upper confidence limit UCL95% < 1 (none occur here). T denotes the region with agex > 35, and S the region with since < 35. Points with dose ≤ 0.5 mGy are omitted

**Fig. 8:**
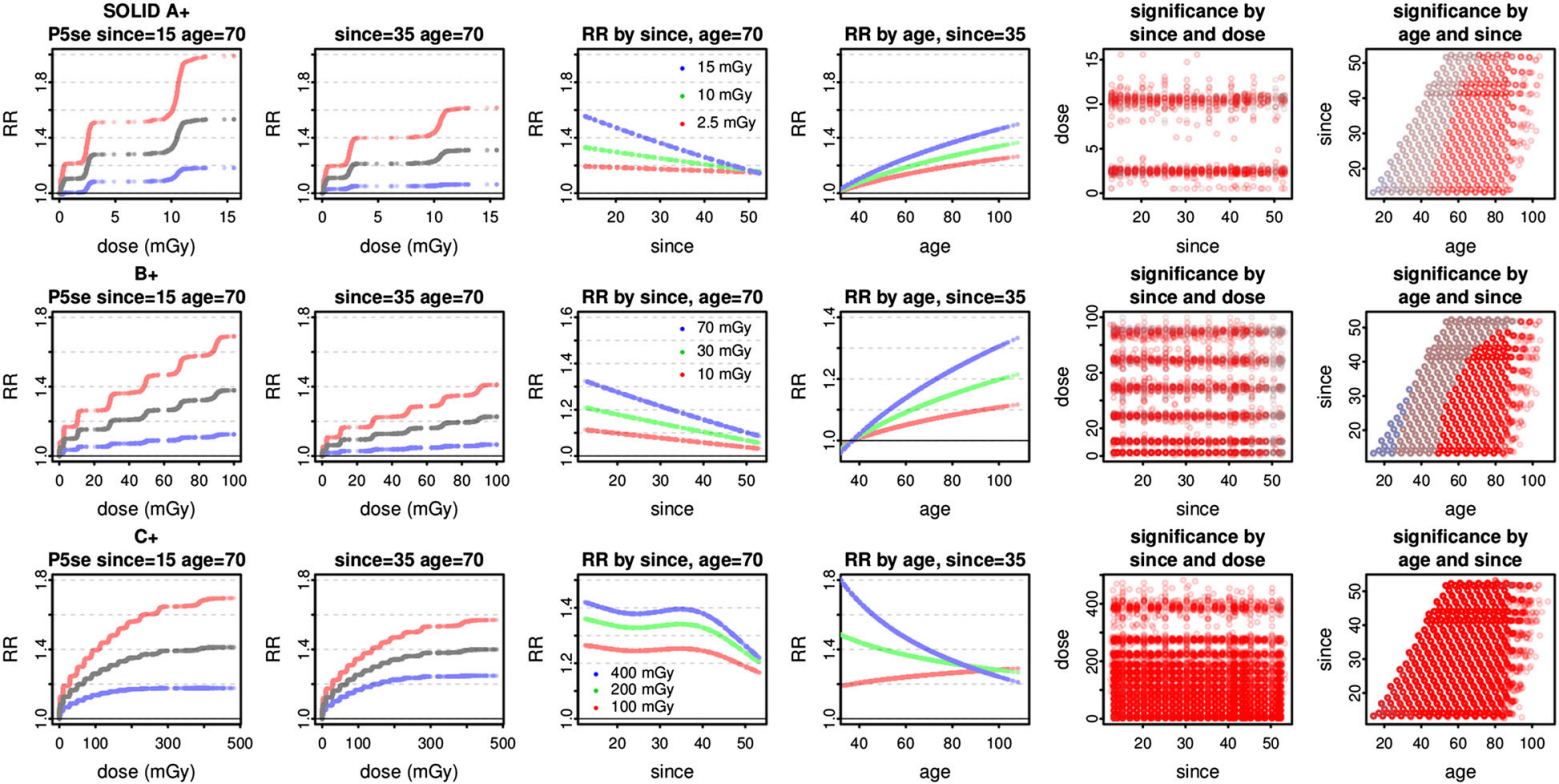
A^+^, B^+^, C^+^ preferred Poisson models, interactions and significance regions. For all solid cancers in A^+^, B^+^, C^+^, the Poisson model P5se (Table 2) minimises ML. RR and 90% CIs are shown at age 70 and since 15 or since 35. RR is plotted against since at age 70, and against age at since 35. Covariate pair shadings are as in Fig. 7, omitting points with dose ≤ 0.5 mGy

Significant elevation of RR persists over a wide range of age and since (or agex), as shown for all models in B^+^ in Fig. 7. With the ecdos models, RR is significantly raised for regions containing age > 55, agex > 35, or since < 35. With the dose models, the regions of significance are similar, though smaller. For the preferred (method 1) Poisson models in A^+^, B^+^, and C^+^, Fig. 8 shows the dose response, the dependence of RR on since and age, and the regions of significance.

The dose models themselves give non-linear responses over C^+^ (see Fig. 3). With P2d, RR rises approximately linearly below 150 mGy, before tapering. Anova comparison with P1d, in which dose appears as a log-linear unsmoothed term, gives p = 0.015. Below 100 mGy, the Excess Relative Risk (ERR = RR - 1) is about 70% higher with P2d than with P1d. With P5sd RR tapers above 150 mGy as with P2d. The term ti(since) has e.d.f. = 3.658 and p = 0.037, and anova(P3d, P5sd) = 0.008 while anova(P4sd, P5sd) = 0.07. Thus P5sd is decidedly non-linear in dose, whose interaction with logage is borderline, but since has significant impact.

The dose models also show RR raised in A^+-^. For P1d and P2d, in both ranges RR |_10 mGy_ = 1.09 (0.99, 1.19). P4sd is preferred amongst dose models by methods 2 and 3. At since 35, in A^-^ RR |_10 mGy_ = 1.11 (1.00, 1.22) with significant elevation when since < 35. In A^+^ RR |_10 mGy_ = 1.09 (0.99, 1.19) with significant elevation when since < 32. In A^+-^ P5sd is preferred amongst dose models by method 1. At age 70, since 35 in A^-^ RR |_10 mGy_ = 1.11 (1.00, 1.23) whilst in A^+^ RR |_10 mGy_ = 1.11 (1.01, 1.22) and RR is significantly raised for 37.2% (28.2%) of cases (py) in D_0.5_, 75.0% (42.3%) in S_0.5_, and 68.4% (79.6%) in T_0.5_.

In general, point estimates and CIs are primarily affected by the dose range (less by the inclusion of NIC) and the choice of dose or ecdos.

The stability of these results was tested, first by using thin plate regression splines. RR and CIs were similar to those shown in Additional file 1: Table S2, but cubic regression splines gave lower ML scores. If exceptional cells detected visually by Cook’s Distance (CD) are deleted, RR values and 90% CIs were slightly altered from those in Additional file 1: Table S2, but still significantly raised (borderline for B^+^ method 2). Deletion affected the choice of preferred models only for C^-^, where P5ae was preferred by all methods, and C^+^, where P5se was preferred by all methods. When preferred models were refitted after excluding cells with the highest 5% of CD values, RR was raised with at least borderline significance across each dose range.

The combined impact of sampling variability and lognormal dosimetry errors was simulated (see Appendix B) using P5se and P5sd in A^-^, shown in Additional file 1: Figure S4. Geometric mean fitted values and 90% CIs are below the values derived from the original data, but still raised with borderline significance; while bootstrap-t [26] and stretched CIs derived from the simulated data remain significantly raised.

Models were fitted with RBE as the function of neutron dose D_n_ shown in [25] (Fig. 4 and Eq. (2)). This raises the dose assigned, as RBE > 50 in the ranges considered here (for which D_n_ < 0.003 Gy). Results are shown in Additional file 1: Table S3. In all dose ranges and with all three methods of Poisson selection, RR is elevated with at least borderline significance. In 15 out of 18 cases, and for 6 of 6 with method 1, the same models are selected, and RR and CIs are slightly lower but still comparable to those in Additional file 1: Table S2.

Excluding genital or, separately, respiratory cancers, the remaining solid cancers were modelled over A^+^, B^+^, and C^+^, with results in Additional file 1: Table S4. RR estimates are mainly similar to those in Additional file 1: Table S2. The heightened significance when agex > 35 or since < 35 generally survives these exclusions.

For urban residents in A, B, or C, the preferred Poisson models show RR elevated with at least borderline significance, and a tapering response at higher doses (see Additional file 1: Table S5). With method 3, RR is significantly elevated in each range.

In each dose range, the factor variables fc and fd have significant interaction, lowering the fitted values for rural Nagasaki residents. Modelling separately by city (Additional file 1: Table S6), for Hiroshima, models with since are preferred for every dose range and selection method. None show RR significantly or borderline raised at rounded mid age and since over A^+-^ or B^+-^, but over C^+-^ RR is significantly raised (method 1) or borderline (at 10 mGy, methods 2 and 3) at age 70 and since 35, and significantly raised (all methods) for high percentages of cases and py.

**Fig. 9:**
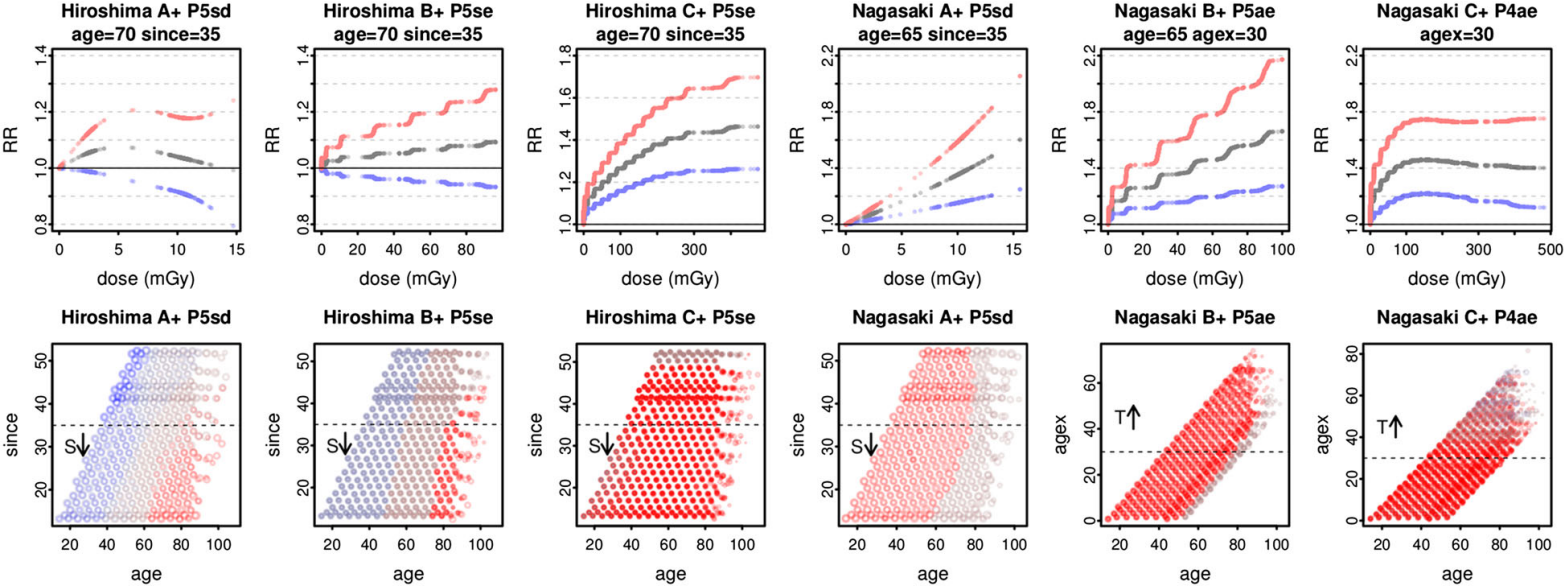
Hiroshima and Nagasaki. For all solid cancers over A^+^, B^+^, C^+^, modelling is carried out separately by city, selecting the Poisson model to minimise ML in each case. In the top row, RR and 90% CIs are shown at the rounded mid age, agex or since as appropriate. In the bottom row, covariate pair shadings are as in Fig. 7, omitting points with dose ≤ 0.5 mGy

For Nagasaki, the preferred models show RR significantly raised in A^+-^ and B^-^ (and for method 1 in B^+^) at rounded mid age, since, or agex, and for high percentages of cases and py. Similar results are obtained over C^+^ with method 1, though exposure at younger ages appears more significant. Figure 9 shows the response by city at the respective rounded mid age and since or agex for the preferred models (method 1) in A^+^, B^+^, and C^+^, and the variation of significance with covariates.

**Fig. 10:**
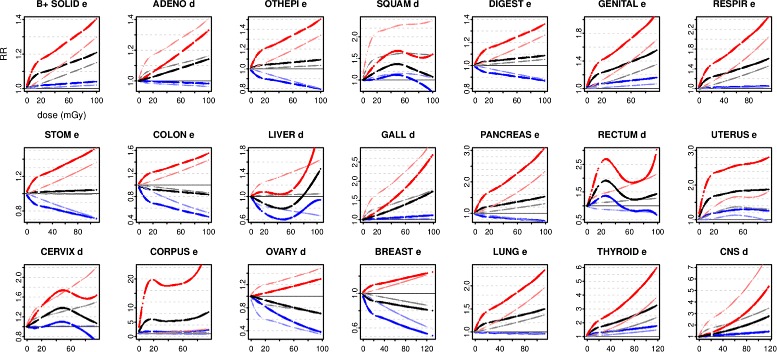
Quasipoisson modelling of specific sites. In B^+^, the simplest quasipoisson models Q2e and Q2d (Table 2) are fitted to all solid cancers and specific sites. In each case, a preferred version is chosen to minimise ML when each model is refitted at the scale optimised for the other member of the pair. The preference is indicated by *e* or *d* in the panel title, and its RR and 90% CI are shown in bold while the unselected model is displayed in the background

Specific sites were analyzed by quasipoisson models with the Pearson estimate of scale $\hat {\phi }$. Additional file 1: Table S7 gives details for Q2 in B^+^ at 21 sites, using scale estimates from Q2e and Q2d with identical values of min.sp. The ML score of Q2d at $\hat {\phi }$_Q2d_ is compared with that of Q2e refitted at $\hat {\phi }$_Q2d_, and vice-versa. The same covariate was preferred whichever optimised scale was used. The Q2 response curves and preferred covariates are shown in Fig. 10. Q2e curves are smoothed for display.

Several sites, including the uterus, show non-linear responses. For all solid, genital, respiratory, gallbladder, thyroid, and cns, the response is significantly elevated for both models in all cells in D_0_. With the preferred covariate, RR is also significantly elevated throughout D_0_ for the uterus and corpus, with weaker evidence for squamous, cervix, and rectum. For lung cancers, using the preferred covariate RR is elevated with borderline significance for over 80% of py in D_0_.

These 12 sites, along with the breast and liver which are radiosensitive at higher doses but show (non-significant) negative response in Fig. 10, were analysed with interaction models. In Additional file 1: Table S8, method 4 is used for each site and dose range, to choose the preferred quasipoisson model. Poisson selection by methods 1, 2, and 3 is also shown. Using the preferred quasipoisson models, RR and 90% CIs are shown along with the percentages of cases and py in D_0_, D_0.5_, S_0.5_, T_0.5_, and S_0.5_ ∩ T_0.5_ for which RR is elevated, significantly elevated, or significantly lowered.

RR is significantly elevated across the dose range for all solid cancers in A^-^, A^+^ (borderline), B^+-^, and C^+-^. Squamous cancers show significantly elevated RR in A^-^, B^-^, B^+^ (borderline 80 mGy), and C^+-^. The corpus has significant, highly elevated RR in A^+-^ and B^+-^. Thyroid RR is significantly elevated in A^+-^, B^+-^, and C^+-^.

Respiratory RR is significantly lowered for 37.2% of py in A^-^ (all with agex < 18), 13.7% of py in A^+^ (all with since > 44) and 11.9% of py in C^-^ (all with agex < 8); it is significantly raised in S_0.5_ and T_0.5_ in B^+-^, and in D_0.5_, S_0.5_ and T_0.5_ in C^+-^. For the breast, RR is significantly lowered only for 10.9% of py in B^-^. There is little significant elevation in A^+-^ or B^+-^, but RR is significantly raised in D_0.5_, S_0.5_ and T_0.5_ in C^-^, and for all py in C^+^. For the liver, RR is significantly lowered for 24.8% of py in A^-^, 26.2% in A^+^, 8.7% in B^-^, 5.6% in B^+^, 10.1% in C^-^, and 2.2% in C^+^.

Additional file 1: Figures S5–S10, for respiratory, uterus, and thyroid, correspond with Figs. 7 and 8 for all solid cancers. For the uterus, RR is a U-shaped function of age. For the thyroid, RR peaks around age 60 and decreases with agex.

Whilst the preferred models vary over the sites, dose ranges, and selection methods, ecdos models are chosen in 215 out of 336 cases, with a similar proportion for each selection method. Models with since are chosen in 194 cases and models with agex are preferred in 80. Among the 24 models for all solid cancers, ecdos is chosen in 22, since in 18, and agex only once. Overall, there is a clear preference for ecdos and since, and models with both covariates are preferred in 140 of 336 cases in total.

The male cohorts in A^+^ and B^+^ were fitted with Poisson models. In A^+^, P5ad (method 1), P4ae (method 2) and P2e (method 3) are preferred. With P5ad at age 70 and with P4ae, the response increases with agex but lacks borderline significance. With P2e the response is insignificant. In B^+^, P5se (method 1), P4se (method 2) and P2e (method 3) are preferred. With P5se at age 70, the response decreases with since but fails to reach borderline significance. With P4se, the response decreases with since and is borderline for since ≤ 20. With P2e, the result is insignificant.

The combined cohorts in A^+^ and B^+^ were fitted with Poisson models, controlling for gender, city, distcat and their first order interactions, obtaining a gender averaged dose response. In A^+^, P5sd (method 1) and P4sd (methods 2 and 3) are preferred. For P4sd at 10 mGy RR is significantly raised (90% CIs) when since ≤ 33. At since 30, RR |_10 mGy_ = 1.09 (1.02, 1.17). Results from P5sd at age 70 are nearly identical. In B^+^, P5se (method 1) and P4se (methods 2 and 3) are preferred. For P4se at since 30, RR |_10 mGy_ = 1.07 (1.01, 1.12), but significance is lost for 19 ≤ since ≤ 27.

## Discussion

Low dose radiation has significant effects at cell level including some non-linear responses [27], but cancer risk is typically modelled as a linear function of dose and the risk below 100 mGy is still under debate. The body’s multiple defences may filter out most abnormalities and the dose-response for disease could be linear.

In analysing the Japanese A-bomb survivors incidence or mortality, the Lifespan studies and other reports [1, 3, 28, 29] use a form of Poisson regression in which ERR = RR - 1 is modelled as *β* *D*, where *β* may be modified by sex, age and age-at-exposure through other parameters; alternatively, ERR ∼*β* *D*+*γ* *D* ^2^. If the response were known to take a specific form with the same parameter values at all doses, a model fitted to the full data could predict risk at 0.01 Gy. Otherwise, a model which estimates risk at 1 Gy may be inappropriate at lower doses, as several studies recognise [2, 8, 30].

In one approach to detecting non-linearity, numerical data can be reduced to categories before smoothing the results [1]. The outcome may depend on the cut points, and information is discarded by categorising data. The Generalized Additive Model [19, 21] is a more sophisticated method. Using GAM, covariates and interactions enter the model through unknown linear combinations of splines.

The public data released by RERF is stratified by essentially arbitrary dose categories with cutpoints 0, 5, 20, 40 mGy …as well as categories of age and age-at-exposure, city, sex and location. However the data also contains py-weighted mean values of dose, age, age-at-exposure, and calendar year (giving time-since-exposure). The analysis uses these values, whose distribution, particularly for dose, is far from uniform. Stratification causes clumping of mean dose, but the individual exposures are also concentrated at low doses. As Preston observed [1] “One aspect of the LSS cohort that is often neglected is the highly skewed dose distribution and the large number of survivors who received low-dose, albeit at a high dose rate, exposure.”

To compensate for stratification and skew, dose was converted to its empirical cumulative distribution, a monotone (invertible) transformation. The resulting variable ecdos is approximately uniformly distributed. Models using ecdos gave better fit for all solid cancers and many specific sites. This transformation may still be relevant with the individual data as dose remains skewed, though no longer clumped.

If the true dose-response is linear then modelling with dose is preferable. Whilst there is little difference in detecting a threshold, ecdos models are better able to capture alternatives, including plateau, hormesis, and a variety of curves with rapid initial rise and a smaller increase at higher doses. One such curve (Fig. 4 panels and) is loosely based on in vivo data for *γ*-H2AX Foci (see [31] (Fig. 1)).

The models have several sources of non-linearity, including the log link, the relation between ecdos and dose, and the splines. In fact, the fitted models are often log-linear in ecdos as a main effect. However, the interaction of ecdos or dose with logage and since or agex can introduce further non-linearities.

For all solid cancers, in A^+-^, B^+-^ and C^+-^ all three Poisson selection methods yield models with RR raised across the dose range at the rounded mid values of covariates, with at least borderline significance. Results are stable when deleting exceptional cells, simulating random errors in dosimetry, adjusting RBE, using different splines, or excluding genital or respiratory cancers. For all solid cancers, results are similar with Poisson or quasipoisson models, which explain around 68% of the deviance in A^+^, 58% in B^+^, and 50% in C^+^.

For all solid cancers in A^+-^, all the dose or ecdos Poisson models show effects which are at least borderline (0.98 ≤ LCL95% ≤ 1) or significant (LCL95% > 1) at 2.5 and 10 mGy, at the rounded mid values of covariates. The preferred models with ecdos refine the results. Significance persists over a wide range of covariates.

With the Nagasaki data, the Poisson model chosen to minimise ML shows RR elevated across the cohort in A^+-^, B^+-^, and C^+^; and in B^+-^ ERR |_10 mGy_ is more than twice the value for the combined cohort. Using a very different approach, Sasaki [7] found an exceptional dose response for Nagasaki (male and female).

For all solid cancers, risk estimates are similar with or without the Not In City data. NIC cells are not distinguished by the urban/rural status of the individual members’ normal residence, which makes a significant contribution to the model. Even without including NIC, extremely low non-zero doses arise from distant rural residents. However, including NIC gives more cases and greater statistical power.

For all solid cancers and most individual sites, time-since-exposure was a better predictor than age-at-exposure. The fitted dependence of risk on since is not governed by a latency threshold, and for all solid cancers, RR decreases with since at low doses. Risk could rise with since < 12.4 years, the minimum value in the cohort.

Shuryak [32] attributes childhood radiation risk to initiation when cells are proliferating, while older adult risk arises from progression of previously acquired mutations. For all solid cancers, RR increases with agex at low doses, suggesting that for women, low dose radiation is acting primarily via progression.

The risks found here are surprising, as linear extrapolation from [1] predicts much lower values at 10 mGy. Preston’s Table 10 shows female ERR = 0.58/Gy with 90% CI (0.43, 0.69) at age 70 and agex 30 for solid cancers in the 0 – 4 Gy (+ NIC) dose range (denoted here as J^+^). Adjusting for age-at-exposure, at agex 35 ERR |_1 Gy_ = 0.53 (0.37, 0.67). Linear extrapolation gives ERR |_10 mGy_ = 0.0053 (0.0037, 0.0067). Over B^+^, the ecdos model P5se is preferred by method 1; at age 70 and since 35 RR |_10 mGy_ = 1.068 (1.018, 1.120), with ERR |_10 mGy_ 12.8 times the extrapolated value. As Table 4 shows, in A^+-^, B^+-^, and C^+-^, the four selection methods choose ecdos models in 22 out of 24 cases, 90% CIs for ERR |_10 mGy_ exclude 0 in those cases and are borderline in 2 cases, and in 23 cases the resulting estimates of ERR |_10 mGy_ are 9.6 – 45.7 times the prediction from linear extrapolation.

**Table 4:** Estimates of ERR at 10 mGy, age 70, agex 35, since 35

Sex	Range	Method	Model	Scale	Covariates	ERR |_10 mGy, Female_	
						90% CI, age 70,	
						agex 35, since 35	Ratio
MF	0–4 Gy		LinExt from [1]	1	s,c,d,dose,age,agex	0.0053 (0.0037, 0.0067)	1.0
MF	0–0.5 Gy (urban)		WtMovAv from [2]	1	s,dose,age,agex	0.02^a^ (estimate)	3.8
F	A^-^	1	P5se	1	c,d,ecdos,age,since	0.242 (0.057, 0.459)	45.7
F	A^-^	2	P4se	1	c,d,ecdos,since	0.225 (0.045, 0.437)	42.5
F	A^-^	3	P4se	1	c,d,ecdos,since	0.225 (0.045, 0.437)	42.5
F	A^-^	4	Q4se	0.901	c,d,ecdos,since	0.228 (0.055, 0.430)	43.0
F	A^+^	1	P5se	1	c,d,ecdos,age,since	0.230 (0.054, 0.436)	43.4
F	A^+^	2	P5se	1	c,d,ecdos,age,since	0.230 (0.054, 0.436)	43.4
F	A^+^	3	P4se	1	c,d,ecdos,since	0.202 (0.032, 0.401)	38.1
F	A^+^	4	Q4sd	0.868	c,d,dose,since	0.088 (-0.001, 0.185)	16.6
F	B^-^	1	P5se	1	c,d,ecdos,age,since	0.088 (0.017, 0.164)	16.6
F	B^-^	2	P4se	1	c,d,ecdos,since	0.085 (0.015, 0.160)	16.0
F	B^-^	3	P4se	1	c,d,ecdos,since	0.085 (0.015, 0.160)	16.0
F	B^-^	4	Q4se	1.141	c,d,ecdos,since	0.077 (0.011, 0.148)	14.5
F	B^+^	1	P5se	1	c,d,ecdos,age,since	0.068 (0.021, 0.117)	12.8
F	B^+^	2	P3d	1	c,d,dose,age	0.013 (0.000, 0.026)	2.5
F	B^+^	3	P2e	1	c,d,ecdos	0.061 (0.015, 0.110)	11.5
F	B^+^	4	Q2e	1.102	c,d,ecdos	0.061 (0.013, 0.112)	11.5
F	C^-^	1	P5se	1	c,d,ecdos,age,since	0.064 (0.038, 0.090)	12.1
F	C^-^	2	P5ae	1	c,d,ecdos,age,agex	0.068 (0.045, 0.091)	12.8
F	C^-^	3	P3e	1	c,d,ecdos,age	0.055 (0.034, 0.076)	10.4
F	C^-^	4	Q3e	1.079	c,d,ecdos,age	0.051 (0.034, 0.068)	9.6
F	C^+^	1	P5se	1	c,d,ecdos,age,since	0.091 (0.055, 0.128)	17.2
F	C^+^	2	P4se	1	c,d,ecdos,since	0.057 (0.040, 0.075)	10.8
F	C^+^	3	P4se	1	c,d,ecdos,since	0.057 (0.040, 0.075)	10.8
F	C^+^	4	Q4se	1.066	c,d,ecdos,since	0.056 (0.039, 0.073)	10.6

*Method* Model Selection Method

*Model* for P5se etc. see Table 2

*LinExt* Linear Extrapolation

*WtMovAv* Weighted Moving Averages

*Covariates* s=sex, c=city, d=distcat

*Ratio* ERR estimate from model / ERR estimate by Linear Extrapolation from [1]

^a^estimate from [2] Fig. 1

However, the GAM Poisson models give estimates comparable with [1] when fitted over J^+^ and evaluated at 1 Gy. Over this range, the preferred model (method 1, 2 or 3) is P5sd. At 1 Gy, age 70 and since 35, RR = 1.80 (1.60, 2.03) while for P5ad at agex 35 RR = 1.56 (1.40, 1.73), close to Preston’s estimate and CI. The dose response in J^+^ for P5ae, P5ad, P5se, or P5sd is roughly comparable with the gender-averaged ERR shown in [1] (Fig. 3, using smoothed category estimates from the 0 – 2 Gy data). Similar results are obtained over J^-^ (excluding NIC).

**Fig. 11:**
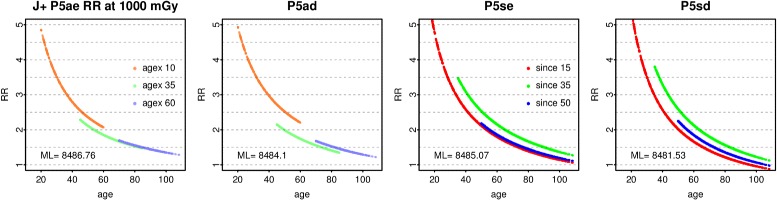
0 – 4 Gy interactions with age at 1 Gy. For all solid cancers in the full female dataset including NIC, as in [1], RR at 1000 mGy is plotted against age for the Poisson models P5ae and P5ad (Table 2), with agex fixed at 10, 35 and 60. Likewise RR at 1000 mGy is plotted against age for the Poisson models P5se and P5sd, with since fixed at 15, 35, and 50

**Fig. 12:**
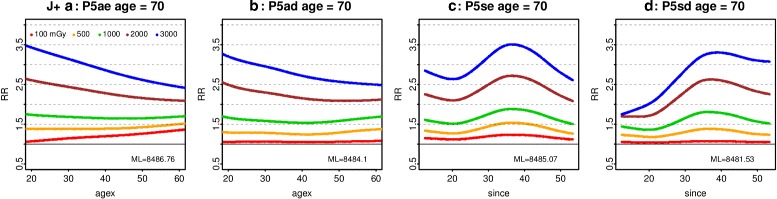
0 - 4 Gy interactions with agex and since. For all solid cancers in the full female dataset including NIC, as in [1], RR at 100, 500, 1000, 2000 and 3000 mGy is plotted against agex (panels **a** and **b**) for the Poisson models P5ae and P5ad (Table 2), with age fixed at 70, and likewise against since (panels **c** and **d**) for the Poisson models P5se and P5sd

At 1 Gy, the interaction of ecdos or dose with logage and agex from fitting P5a over J^+^ is consistent with [1], whose Fig. 4 is comparable with Fig. 11 here. If agex or since is fixed, RR |_1 Gy_ decreases with age. Preston’s Fig. 6 (using smoothed category estimates) is comparable with the curve for 1000 mGy in Fig. 12 panel (P5ad at age 70). At age 70, RR |_1 Gy_ initially decreases with agex but rises when agex > 40. Using P5sd, with better fit, at age 70 RR |_1 Gy_ peaks at since 35. Similar curves arise at age 50, but with higher values of RR.

However, the interactions at lower doses (see Figs. 5, 6, and 8) conflict with the main model in [1], which has RR decreasing with agex. At low doses, RR generally decreases with age when agex is fixed, but increases with agex when age is fixed. Likewise, above 100 mGy in C^+^ RR decreases with age when since is fixed, but increases with age in A^+^ and B^+^. At age 70, RR decreases with since in A^+^ and B^+^ and when since > 35 in C^+^. In all the low dose ranges considered here, RR increases with agex. Even in J^+^, the curve for 100 mGy does not decrease with agex (see Fig. 12 panel) Thus, the decrease of RR with agex (when agex < 40) in [1] is specific to the higher dose range and the response at 500 mGy or above.

Pierce and Preston [2] applied a simplified linear model to the 0 – 500 mSv urban incidence data for both sexes, finding a higher response than linear extrapolation would predict. Their Fig. 1 shows a smoothed curve obtained as a weighted moving average of category estimates. The sex-averaged RR at agex 30 and 100 mSv ∼ 1.09, from which RR |_female, 100mSv_ ∼ 1.12 as the fitted model has ERR |_female_ ∼ 2*ERR |_male_. At agex 35, RR |_female, 100mSv_ ∼ 1.10. Figure 1 suggests that RR |_female, 10mSv_ ∼ 1.02. By comparison, the preferred model (method 1) for the urban subcohort of C^-^ is P5se, and at age 70, since 35, RR |_100 mGy_ = 1.14 (1.09, 1.18), while RR |_10 mGy_ = 1.03 (1.02, 1.04). The interaction with since is very weak, and the preferred model with method 2 or 3 is P3e, for which at age 70 RR |_100 mGy_ = 1.14 (1.09, 1.19), as with the preferred quasi model Q3e. Models with either dose or ecdos show RR rising rapidly below 150 mGy before tapering, evidence of non-linearity in the urban data below 500 mGy.

**Fig. 13:**
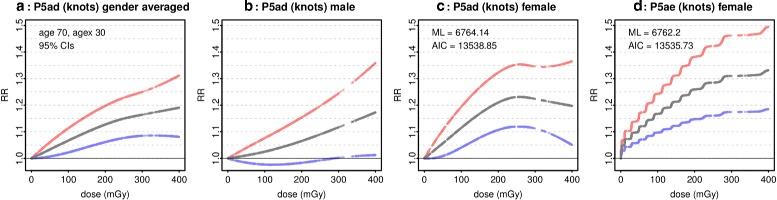
0 - 400 mGy male and female. For comparison with [8], panel **a** shows the dose model P5ad (Table 2) fitted to the 0 - 400 mGy male-female data, with knots set at 0, 5, 20, 40, 60, 80, 100, 125, 150, 175, 200, 250, and 300 mGy. Gender averaged RR and 95% CIs are shown at age 70 and agex 30. Panels **b** and **c** show the male and female responses. Panel **d** shows the ecdos model P5ae with corresponding knots at ecdf(dose)(0), ecdf(dose)(5) etc. ML scores and AIC for P5ad and P5ae fitted to the female data are shown for comparison

Furukawa and co-workers [8] analysed the combined male and female incidence data below 400 mGy with models including Linear Non Threshold, cubic smoothing spline, and a Bayesian semiparametric piecewise linear model, with dose responses at age 70, agex 30 shown in their Fig. 1. The Bayesian and spline models gave similar responses at low doses. Whilst comparisons with the Bayesian model are beyond the scope of this paper, the spline model is roughly similar to P5ad, if dose knots are set at 0, 5, 20, 40, 60, 80, 100, 125, 150, 175, 200, 250, and 300 mGy. Furukawa analysed the individual data, but results using P5ad (with these knots) applied to the public data below 400 mGy are not significantly different. Figure 13 (panel) shows the gender averaged dose response for P5ad (knots). The spline estimate in [8] for ERR |_100 mGy_ = 0.03 (CIs are not shown for this model) whilst for P5ad (knots) ERR |_100 mGy_ = 0.07 (0.02, 0.11)_95%_. The difference might result from the interaction structures in [8] which follow those in [1]. However, as Fig. 13 shows, the male response (panel) lacks 95% significance below ∼ 300 mGy, but (panel) female ERR |_100 mGy_ = 0.11 (0.04, 0.19). Panel shows the ecdos model P5ae with knots corresponding to those chosen for P5ad (knots). Now ERR |_100 mGy_ = 0.17 (0.10, 0.24). With the female data, P5ae (knots) is preferred to P5ad (knots) by ML score and AIC. At 10 mGy with 95% CIs, P5ad (knots) gives ERR |_10 mGy_ = 0.011 (-0.001, 0.023) whilst for P5ae (knots) ERR |_10 mGy_ = 0.052 (0.031, 0.074).

The values listed in Table 4 are obtained from preferred models. P5ad is never selected, and P5ae is chosen only once. Table 4 largely reflects the predominant selection of ecdos models. Nevertheless, if P5ad and P5ae (with their default knot locations) are fitted over A^+^ and evaluated at 10 mGy, age 70 and agex 35, for P5ad RR = 1.10 with 90% CI (1.00, 1.21) while with P5ae RR = 1.21 (1.04, 1.41). P5ae is again preferred to P5ad by ML score and AIC. The default knots for P5ad in A^+^ are 0, 0.12, 0.33, 2.30, 2.57, 9.69, 10.55, 15.58, giving a much richer structure than 0, 5, 20. The estimate at 10 mGy when fitting P5ad over A^+^ is much higher than the estimate when fitting P5ad over C^+^.

Preston and co-workers [33] analysed the 1958–1998 solid cancer incidence data for exposure in utero or early childhood (0 – 5 years), including NIC. For women exposed in early childhood, at attained age 50 ERR |_1 Sv_ = 2.2 (1.3, 3.4)_95%_ (see [33] Table 5). Linear extrapolation is comparable with results from P5sd over C_i_^+^ (agex ≤ 5, dose ≤ 500 mGy) at age 50 and since 45, for which RR |_10 mGy_ = 1.03 (1.01, 1.05)_95%_. With the preferred Poisson model (method 1) P5se, RR |_10 mGy_ = 1.20 (1.06, 1.35)_95%_. Methods 2 or 3 select P2e, giving RR |_10 mGy_ = 1.14 (1.05, 1.24)_95%_, while method 4 chooses Q2e giving RR |_10 mGy_ = 1.14 (1.06, 1.22)_95%_.

For site-specific risks, some studies of other cohorts have found RR significantly raised after low dose exposure to low LET radiation. Some evidence from the literature on the thyroid, uterus and uterine corpus, is compared with results from quasipoisson models applied to appropriate subcohorts here.

Direct evidence on thyroid cancer risk arises from children given medical irradiation, residents affected by Chernobyl, nuclear workers, radiotherapy patients, and the A-bomb survivors. Except as indicated, these studies applied a linear ERR model.

Additional file 1: Table S9 shows some results from [1, 6, 7, 11, 13, 15, 34–37]. In each case, an appropriate subcohort of the female incidence data was analysed for comparison, using the simplest quasipoisson dose model Q2d and the preferred quasi model for the subcohort, chosen to approximate the dose and agex range in the study, or with mean dose close to that at which risk was reported.

In most cases, results from the subcohorts using either model are compatible with the reported estimates, as CIs overlap. There is striking agreement with the *tinea capitis* studies. Sadetzki [34] used a linear model which, at the mean dose, gives RR |_93 mGy, female_ = 2.96 (2.07, 4.31)_95%_. Fitting the quasi models over C^+^ restricted to agex < 16, Q2d gives RR |_93 mGy_ = 2.30 (1.36, 3.89)_95%_, while the preferred model Q2e gives RR |_93 mGy_ = 2.95 (2.05, 4.24)_95%_. The earlier study by Ron [35] included the estimate RR |_10 mGy_ = 1.27 (1.15, 1.42)_95%_. Fitting the quasi models over B^+^ restricted to agex < 16, for which the mean py-weighted dose = 10.7 mGy, Q2d gives RR |_10 mGy_ = 1.18 (1.06, 1.31)_95%_, and at the mean values of agex and age in [35] the preferred model Q5ad gives RR |_10 mGy, agex 7.4, age 37.6_ = 1.28 (1.07, 1.53)_95%_. These close agreements contrast with the differences seen in the pooled study [11], where the *tinea capitis* estimates appeared exceptionally high. However, the pooled estimates are compatible with GAM modelling on the appropriate subcohorts.

For Chernobyl survivors with agex < 18, Little [6] considered a linear-exponential model with low-dose asymptote ERR = *α*D, estimating *α* = 9.72 (2.67, 94.31)_95%_. At the geometric mean dose as reported in [38], this gives an asymptotic RR |_190 mGy_ = 2.85 (1.51, 18.92)_95%_. Fitting the quasi models over C^+^ restricted to agex < 18, Q2d gives RR |_190 mGy_ = 2.62 (1.50, 4.57)_95%_. At the mean attained age from [39], the preferred model Q3e gives RR |_190 mGy, age 23.7_ = 4.27 (1.30, 14.02)_95%_.

Sasaki [7] reanalysed the incidence data in [1], with a very different non-linear model. For the thyroid, from his Fig. 3(i) ERR |_0.1 mSv, female_ ∼ 0.35, though no CI or interactions are shown. Results from C^+^ appear compatible. The occupational study by Sont [13] did not estimate RR for female thyroid cancer, but found a Standardized Incidence Ratio = 1.42 (1.19, 1.69)_90%_. This was comparable with internal estimates of SIR (see “Methods”), with CIs by non-parametric bootstrap.

The results from GAM models applied to relevant subcohorts are compatible with [6, 11, 13, 15, 34, 35]. They are higher than extrapolations from [1, 36, 37], which concerned higher dose ranges. However they appear compatible with [7].

Several studies found RR decreasing at higher doses. In the radiotherapy studies, doses were high and the downturn was only apparent above 6 Gy [5] or 10 Gy [36], whilst for Chernobyl survivors risk tapered above 1 Gy [6]. Using the no-interaction dose model Q2d, RR tapers above 400 mGy.

Unlike the thyroid, the uterus is not generally recognised as radiosensitive, although [1] notes an excess of cancers of the uterine corpus at young ages. Occupational studies [40–42] found significant excess mortality from uterine cancers in nuclear workers with a radiation record. The mortality rate increased with dose, but with few cases, mainly endometrial (corpus), ERR/Sv was not reported.

Analysing the National Registry for Radiation Workers, Muirhead [43] found that for mortality (internally calculated SMR) amongst solid cancers, only all uterine cancers showed trend with dose significant at the 5% level using a two-sided test. For incidence, there was a significant increasing trend with dose for endometrial cancer. RR was estimated with a linear model. For incidence of uterine ca ERR/Sv = 10.523 (0.27, 39.4)_90%_. Near the mean dose (24.9 mSv), this corresponds to RR |_25 mSv_ = 1.26 (1.01, 1.99). For incidence of corpus ca, the Health Protection Agency [44] reported that Muirhead found ERR/Sv = 14.2 (1.06, 58.56)_90%_, corresponding to RR |_25 mSv_ = 1.36 (1.03, 2.46).

For comparison, models were fitted over B^+^, restricted to 16 ≤ agex < 60. For the uterus, Q2d gives RR |_25 mGy_ = 1.07 (0.99, 1.15). The preferred model Q3d has RR |_25 mGy, 60yrs_ = 0.96 (0.75, 1.23), while at age 65 RR = 1.23 (0.98, 1.54), and at age 70, 1.29 (1.04, 1.60). For the corpus, Q2d gives RR |_25 mGy_ = 1.08 (0.89, 1.31). The preferred model Q4se gives RR |_25 mGy, since 30_ = 5.24 (1.22, 22.55), and 4.90 (1.13, 21.24) and 4.00 (0.93, 17.14) at since 25 and 35 respectively.

Davis [16] studied solid cancer incidence in the Techa River cohort, and found significant effects only for the oesophagus and uterus, using a linear model without interactions. Women constitute 57% of the cohort and age of entry begins at 0. The mean dose was 53 mGy and 90% of doses were below 125 mGy. For the uterus, mean age at diagnosis was 57, and the result for ERR/100mGy = 0.21 (0.01, 0.51)_95%_ corresponds to RR |_50 mGy_ = 1.11 (1.01, 1.26)_95%_.

For comparison, models were fitted over B^+^. Q2d gives RR |_50mGy_ = 1.37 (1.09, 1.72)_95%_. The preferred model Q5se gives RR |_50mGy, age 60, since 15_ = 1.96 (1.12, 3.42)_95%_, and at since = 20, 25, 30, and 35, the corresponding RR and 95%CI = 1.82 (1.10, 3.02), 1.67 (1.01, 2.76), 1.64 (0.99, 2.73), and 1.70 (1.02, 2.84).

Using a linear model without effect modification, Preston [1] estimated for the uterus ERR/Gy = 0.10 (-0.09, 0.33)_90%_, and for the corpus ERR/Gy = 0.29 (-0.14, 0.95). However, for women with agex < 20, ERR/Gy for the uterus = 0.37 (0.001, 0.86) and for the corpus 1.00 (0.14, 2.40). Near the py-weighted mean dose (85.3 mGy), with agex < 20 the model predicts for the uterus RR |_80 mGy_ = 1.03 (1.00, 1.07)_90%_ and for the corpus 1.08 (1.01, 1.19).

Fitting over C^+^ restricted to agex < 20, for the uterus Q2d gives RR |_80 mGy_ = 1.79 (1.19, 2.68)_90%_. With the preferred model Q5se, RR |_80 mGy, age 50, since 30_ = 2.64 (1.17, 5.97)_90%_, and at since = 35, 40, 45 and 50 the corresponding RR and 90%CI = 3.07 (1.41, 6.67), 4.51 (2.05, 9.94), 3.76 (1.65, 8.59), and 2.72 (1.11, 6.62). For the corpus Q2d gives 1.03 (0.88, 1.21). For the preferred model Q4ad, RR |_80mGy, agex 10_ = 0.88 (0.56, 1.37), whilst at agex 12, 14, 16, 18 and 20 the corresponding values are 1.13 (0.74, 1.74), 1.59 (1.05, 2.43), 2.04 (1.33, 3.11), 2.24 (1.40, 3.60), and 2.18 (0.96, 4.98), i.e. the response is higher after puberty.

I have not used another implementation of GAM or the fully Bayesian methods of MCMC. Confidence intervals, based on Wald-type CIs for the linear predictor, were validated by simulation only for Poisson modelling of all solid cancers. Although the models explain nearly 60% of the deviance for all solid cancers (in B^+^), the figures for individual cancer sites are lower, given the smaller case numbers. Minimum smoothing parameters imposed for some sites depended on a visual judgement of the output. The public data lacks information on other exposures which may interact with radiation. The effects could be due to confounding by some other factor linked to distance from the hypocentres, although modelling did control for urban, rural, or NIC location. The modelling was not designed to test a particular hypothesis for cellular mechanism, or for the higher risks for women or Nagasaki residents.

Although increased risk with age-at-exposure (at fixed attained age) was found for many sites, as Boice observed [45] the flash doses all occurred in August 1945, so age-at-exposure is confounded with birth cohort and changes in diet, smoking habits or infection rates, may explain apparent age-at-exposure (or time-since-exposure) effects. Protracted or fractionated exposures may also have different effects.

The impact of radiation in this cohort may be affected by blast damage. Stewart and Kneale [46] investigated possible selection bias, as subjects were only eligible to enter the (mortality) cohort if they survived to 1950. Incidence data begins in 1958, with the minimum time-since-exposure exceeding 12 years.

Wing and Richardson [47] point out that the dose estimates, revised several times, also depend on “the ability to elicit accurate information on location, position and shielding [which] was affected not only by traumatization of the survivors and their domestic stigmatization but by their distrust of medical teams working under occupation forces [48].” The A-bombs annihilated Hiroshima and Nagasaki, destroying their social fabric.

For all these reasons this cohort may not predict risk elsewhere.

## Conclusion

The Generalized Additive Model avoids potential problems in fitting a mis-specified parametric model. In fact, the linear model is an inadequate description of relative risk for cancer incidence in female Japanese A-bomb survivors exposed to low dose radiation. Contrary to the interactions at high doses, for all solid cancers risk increases with age-at-exposure and decreases with time-since-exposure, suggesting that low dose radiation acts through progression of previously accumulated damage.

Time-since-exposure models were generally preferred to age-at-exposure models. Transforming dose to its empirical cumulative distribution improved the model fit for all solid cancers and many specific sites. Constructing splines in this way improves their capacity to detect responses at very low doses. Covariate interactions were modelled with tensor product splines. Marginal Likelihood was the preferred method of smoothing parameter optimisation, and confidence intervals obtained from the Bayesian posterior covariance performed well.

The results at 10 mGy are much higher than expected. Whilst higher female cancer risks and the highly skewed distribution of dose are recognised, significantly raised Relative Risk for women exposed to low doses, not found for all solid cancers in men, is new. Most other A-bomb survivor studies consider much wider dose ranges.

Whilst the results may be specific to this cohort, some studies of other cohorts with other methods give comparable results, notably for thyroid cancer where estimates are consistent with those derived here, and for the uterus.

Direct epidemiological evidence of risks from doses around 10 mGy is a current research priority [18]. Risk estimates derived here from low dose data are much higher than those on which the ICRP has relied when setting recommended annual dose limits [4], including the annual occupational dose limit of 20 mSv. For all solid cancers, almost all estimates here of Excess Relative Risk for women at 10 mGy are 9.6 – 45.7 times the prediction by linear extrapolation from [1].

Generalized additive models applied to the low dose data for female Japanese A-bomb survivors show significant Relative Risks, particularly if the heavily skewed doses are transformed to improve their distribution. Evidence of elevated risk at low doses should contribute to higher radiation risk estimates and improve radiation protection for women.

## Additional file

Additional file 1 Additional files are linked from a mini-website available with the online version of this paper. They consist of 1 PDF file with the Appendices, 10 images, 1 Excel workbook with 9 tables, 4 code files, 2 data files, and 6 output files. (ZIP 3727 kb)

## Abbreviations

*χ*^2^: Pearson Chi-squared statistic
$\hat {\phi }$: Pearson estimate of scale for quasipoisson model
A^+-^: 0 - 20 mGy including (excluding) NIC subjects
B^+-^: 0 - 100 mGy +- NIC
C^+-^: 0 - 500 mGy +- NIC
CD: Cook’
s Distance; CI: Bayesian posterior confidence interval
D_*δ*_: data cells with dose >
*δ*; dfres: residual degrees of freedom
e.d.f.: effective degrees of freedom
ERR: Excess Relative Risk
ecdf: empirical cumulative distribution function
ecdos: empirical cumulative distribution of dose
GAM: Generalized Additive Model
GCV.Cp: Generalized Cross Validation/Mallows Cp
J^+-^: 0 - 4 Gy +- NIC
LCL: Bayesian posterior lower confidence limit
ML: Marginal Likelihood
NIC: Not In City
REML: Restricted Maximum Likelihood
RERF: Radiation Effects Research Foundation
RR: Relative Risk
S_*δ*_: dose >
*δ* and since < 35; T_*δ*_: dose >
*δ* and agex > 35; UCL: Bayesian posterior upper confidence limit
