# Supplementary material for: Low dose radiation risks for women surviving the a-bombs in Japan: generalized additive model
Source: Environ Health. 2016 Nov 24;15:112. doi: 10.1186/s12940-016-0191-3 (PMC5121957; doi:10.1186/s12940-016-0191-3)
Supplement: Additional file 1 — Additional files are linked from a mini-website available with the online version of this paper. They consist of 1 PDF file with the Appendices, 10 images, 1 Excel workbook with 9 tables, 4 code files, 2 data files, and 6 output files. (ZIP 3727 kb) [file 12940_2016_191_MOESM1_ESM.zip › appen.pdf]

Appendix A

Relative Risk RR and its confidence intervals are obtained from a prediction matrix and fitted coefficients. A model M together with a data frame D of covariate values generates a prediction matrix P which, when multiplied with the fitted coefficient vector $\hat{\beta}$ yields the linear predictor for D excluding the offset: $\hat{\eta} = \mathbf{P}\hat{\beta}$. The fitted values are recovered as predict(M,D) $= exp(log(py)+\hat{\eta}) = py*exp(\hat{\eta})$, given the log link and offset of log(py). The fitted estimate of risk in each cell is predict(M,D)/py $= exp(\hat{\eta})$. A new data frame D_0 is generated with the ecdos values replaced by their minimum, attained when dose = 0, i.e. ecdos=ecdf(dose)(0), giving the corresponding linear predictor $\hat{\eta}_0 = \mathbf{P_0}\hat{\beta}$. For dose models, D_0 is obtained by setting dose = 0. In each cell, Relative Risk RR is defined as (predict(M,D)/py)/(predict(M,D_0)/py) $= exp(\hat{\eta} - \hat{\eta}_0)$. Note that the baseline estimates, predict(M,D_0), are obtained after fitting, and in each cell RR = fitted value / fitted baseline. As log(RR) = $(\mathbf{P}\text{-}\mathbf{P_0})\hat{\beta}$, Cov(log(RR)) = $(\mathbf{P}\text{-}\mathbf{P_0})\mathbf{V}(\mathbf{P}\text{-}\mathbf{P_0})^T$ where $\mathbf{V}$ is the covariance matrix of $\hat{\beta}$, and $(diag[(\mathbf{P}\text{-}\mathbf{P_0})\mathbf{V}(\mathbf{P}\text{-}\mathbf{P_0})^T])^{1/2}$ gives a vector sd of standard deviations for log(RR). The default estimator of $\mathbf{V}$ in mgcv is the Bayesian posterior covariance (see [1] Ch 4). The 90% CI for RR is (LCL95% , UCL95%) with endpoints $exp(\hat{\eta} - \hat{\eta}_0 \mp sd * qnorm(0.95))$. Note that RR and CIs are defined in each cell.

For P2, RR $= exp(\hat{\eta}-\hat{\eta}_0)$ depends only on ecdos (or dose), and the above approach leads to plots of RR and its confidence intervals against ecdos (or dose). With the interaction models, the dose-response is a surface from which curves are obtained by fixing the value of other relevant covariates. For example with P4a, ecdos (or dose) interacts with agex and $RR|_{agex=35}$ is the risk at agex 35, divided by the risk at agex 35 with dose 0, after fitting the model over all data. To evaluate it, all values of agex in D and D_0 are reset to 35. $RR|_{agex=35}$ depends only on ecdos (or dose). To view $RR|_{dose=50}$ as a function of agex, set ecdos=ecdf(dose)(50) or dose=50 in D. The same methods are used for the corresponding quasipoisson models.

The opening section of Additional File code1.txt, prior to the simulations, illustrates these techniques for the Poisson model P4se.

For model selection, Method 1, for Poisson only, chooses the model with lowest ML score. This mild approach is only appropriate for quasipoisson models if all have the same scale. Method 2, for Poisson only, selects the model with lowest AIC. In method 3, for Poisson only, each pair of nested models is compared by anova (test=Chisq). Forward and backward selection is conducted starting with P2. The larger model is rejected if the anova $p > 0.05$. Anova can fail, as Dev may increase or e.d.f. decrease for the larger model (fitting uses ML, not Dev, and optimising over a larger function space may find a model with lower e.d.f.). If anova fails, and for all non-nested comparisons, the model with lower AIC is preferred in that pair.

In method 4, for quasipoisson, anova is conducted with test=F as scale estimates vary, and proceeds as in method 3 until anova fails. AIC is unavailable, and the ML score is sensitive to scale. Instead, M2 is refitted at the optimised scale sc1 for M1 and anova is repeated on M1 $\subset$ M2 with test=Chisq, and likewise with M1 refitted at scale sc2 for M2. If anova still fails or gives conflicting results at sc1

and sc2, the model with lower ML score (normally M2) when the pairs are refitted at sc1 is chosen, and likewise at sc2. For example if e.d.f. is lower with the larger model, anova fails but it is appropriate to select M2 if ML has decreased. If the choice still depends on which optimised scale was used, pick the model with lower e.d.f.. For non-nested pairs, models are refitted at sc1 and sc2 and ML scores are compared, and if the choice depends on the optimised scale, choose the model with lower e.d.f..

Appendix B

To choose and validate the smoothing parameter optimisation method, the Bayesian posterior confidence intervals for a Poisson model M with fitted values F were simulated 1000x. Put obsx <- rpois(L,F) where L=length(obs) is the number of records and rpois draws from a Poisson distribution with parameter vector F.

Bootstrap-t CIs of log(RR) were obtained at quantiles of the dose, with other interaction covariates fixed at their rounded mid value. For each replication, the simulated data obsx is refitted by M and $\log(\mathrm{RR})^* = (\hat{\eta} - \hat{\eta}_0)^* = ((\mathbf{P}\text{-}\mathbf{P_0})\hat{\beta})^*$ is evaluated along with the corresponding standard deviation derived from the covariance $((\mathbf{P}\text{-}\mathbf{P_0})\mathbf{V}(\mathbf{P}\text{-}\mathbf{P_0})^T)^*$, when ecdos = (j-0.5)/10 for j=3,4,. . . 10, with rounded mid since or agex and/or age as appropriate. For each j, this gives a t-statistic

$$\frac{((\mathbf{P}\text{-}\mathbf{P_0})\hat{\beta})^* - ((\mathbf{P}\text{-}\mathbf{P_0})\hat{\beta})}{((diag[(\mathbf{P}\text{-}\mathbf{P_0})\mathbf{V}(\mathbf{P}\text{-}\mathbf{P_0})^T])^{1/2})^*}$$

whose distribution leads to bootstrap-t confidence intervals [2] for $\log(\mathrm{RR}) = \hat{\eta} - \hat{\eta}_0$ at the ecdos values. These CIs are exponentiated to give CIs for RR.

Coverage was estimated for the Bayesian posterior upper and lower CIs, which were each stretched (or shrunk) to achieve an average 97.5% coverage across the dose quantiles. The bootstrap-t and stretched CIs for dose quantiles are shown on the Bayesian posterior CI plots, for comparison. See Additional File code1.txt.

Alternatively, cross validation was used for the distributions of $\mathrm{RR}|_{10\ \mathrm{mGy}}$ and the proportion of deviance explained. Half the records were selected as a training set by random sampling without replacement. The model was fitted using only the selected rows S, and the fitted model used for prediction in the unselected rows nS, leading to predicted $\mathrm{RR}|_{10\ \mathrm{mGy}}$ (with interaction covariates fixed at their rounded mid values). Repeating 1000x yields a distribution of predicted $\mathrm{RR}|_{10\ \mathrm{mGy}}$.

Fitting the model M over the full data gives the proportion of deviance explained, devex = (deviance(M_0)–deviance(M))/deviance(M_0) where M_0 is the null model obs $\sim$ offset(log(py)). Deviance depends only on the observed and fitted values, and fitted(M_0) = py*sum(obs)/sum(py). Fitting M over S gives devexS, the proportion of deviance explained over S. The predicted proportion of deviance explained, devexnS, is evaluated using the observed and predicted values over nS. Repeated sampling gives distributions devxS and devxnS for comparison with each other and the original value devex. For details see Additional File code2.txt.

The ability of ecdos and dose models to detect potential dose-responses was simulated. Suppose $ERR = f(dose, \beta, \sigma, \tau)$ is the presumed Excess Relative Risk with specified function and parameters. The initial model m_0 is fitted over B^- and new data simulated with RR = 1+ERR, by sampling obsx <- rpois(L,μ_0*(1+ERR)) where L = length(obs) and μ_0 are the fitted m_0 values. This generates data with μ/μ_0 = RR. Model with P2e (using ti(ecdos)) and P2d (using ti(dose)) to give fitted estimates RR* at 10, 20 ...100 mGy with confidence limits at each dose. Repeating 500x form the 500x10 matrix C_e whose [k,j] element = 1 if, for the kth simulation fitted by P2e the CI at dose=10*j contains $RR|_{dose=10*j}$ and 0 otherwise; likewise form C_d using P2d. Coverage averaged over the dose range is estimated as mean(C_e) and mean(C_d). At each dose=10*j, geometric means of the 500 simulated RR*, lower and upper confidence limits give an estimated average result of fitting P2e or P2d to data generated from the presumed RR. See Additional File code3.txt.

The combined impact of sampling variability and lognormal dosimetry errors with geometric mean 1 and geometric standard deviation 1.2 was simulated. Put obsx <- rpois(L,F) as previously, define dose1 <- rd*dose where rd <- exp(log(1.2)*rnorm(L)), and ecdos1 <- ecdf(dose1)(dose1). Refit the model using obsx and ecdos1, giving RR* and confidence limits lo* and hi*, all evaluated at quantile(dose, (j-0.5)/10) j=1,2...10, i.e. at doses from the original data, and rounded mid-values of interaction covariates. The geometric means of RR*, lo* and hi* are reported with bootstrap-t and stretched CIs. See Additional File code4.txt.

References

[1] Wood, S.N.: Generalized Additive Models: An Introduction with R. Chapman & Hall/CRC; Taylor & Francis Group, Boca Raton, London, New York (2006)

[2] DiCiccio, T.J., Efron, B.: Bootstrap confidence intervals. Statist Sci **11(3)**, 189–228 (1996)
