# Supplementary material for: Low dose radiation risks for women surviving the a-bombs in Japan: generalized additive model
Source: Environ Health. 2016 Nov 24;15:112. doi: 10.1186/s12940-016-0191-3 (PMC5121957; doi:10.1186/s12940-016-0191-3)
Supplement: Additional file 1 — Additional files are linked from a mini-website available with the online version of this paper. They consist of 1 PDF file with the Appendices, 10 images, 1 Excel workbook with 9 tables, 4 code files, 2 data files, and 6 output files. (ZIP 3727 kb) [file 12940_2016_191_MOESM1_ESM.zip › index.html]

Supplementary Files


## Supplementary Files

|  |
| --- |
| *The supplementary files linked below consist of 1 pdf file with the Appendices, 10 images (\*.tif), 1 Excel workbook (\*.xls) with 9 tabs, 4 text-only (\*.txt) code files, 2 text-only data files, and 6 text-only output files.*  To run the codes, follow instructions below for saving into a new folder "mini" within your main Documents folder. Data files are required, and Output files are optional when running code.  Appendices  Images  Tables  Codes  Data  Output Appendices Appendix A and Appendix B Images Figure S1  Dose response for all solid cancers in B+, using Poisson ecdos and dose models from P2e and P2d upwards (see Table 2 in main text for model definitions). Relative Risk and Bayesian posterior 90% CIs are shown at rounded mid values of age, age-at-exposure, or time-since-exposure as appropriate.  Figure S2  For all solid cancers in A+-, B+-, and C+-, the ratio of influence of P5sd (Table 2) to influence of P5se is evaluated at each cell. Influence values appear on the diagonal of the influence matrix of the fitted model. The ratio is plotted against dose (black dots) and a smoothing spline (red) fitted to the result.  Figure S3  For all solid cancers in B+, P2d and P2e are as in Table 2. P2g is constructed similarly using gdos, a smooth approximation to ecdos shown in panel **a**, using the formula displayed with parameters α = 0.148, β = 0.0062, σ = 0.247, τ = 0.465. Panel **b** shows RR and 90% CIs from P2e and P2g. Panels **c**, **d** and **e** show the first 3 splines constructed from dose, ecdos, and gdos.  Figure S4  For all solid cancers over A-, the Poisson model P5se (Table 2) which minimises ML, and P5sd which does so amongst dose models, are fitted and RR and 90% CIs are displayed as usual. Simulation at age 70 and since 35 (similar to Figure 2 in the main text) is extended to allow random errors in dosimetry, with a log-normal distribution with Geometric Standard Deviation = 1.2. Bootstrap-t CIs (solid triangles) and stretched CIs (open circles) are shown along with geometric means (open triangles) of the simulated RR (green) and Bayesian posterior 90% CIs (blue and red), for comparison with the Bayesian CIs from the original data. See Appendix B.  Figure S5  For respiratory cancers in B+, quasipoisson ecdos and dose models (Table 2) are plotted on the top and bottom rows respectively. RR and 90% CIs are shown for Q2e and Q2d. For the interaction models, covariate pairs are shaded red if the lower confidence limit LCL95% > 1, brown if RR > 1 but LCL95% ≤ 1, blue-grey if RR ≤ 1 but UCL95% ≥ 1, and bright blue if the upper confidence limit UCL95% < 1. T denotes the region with agex > 35, and S the region with since < 35. Points with dose ≤ 0.5 mGy are omitted.  Figure S6  For respiratory cancers in A+, B+, C+, the preferred quasipoisson model is selected in each dose range. RR and 90% CIs are shown at since 15 or 30, or at agex 45 or 20. RR is plotted against since, and against age. Shadings are as in Figure S5. Points with dose ≤ 0.5 mGy are omitted.  Figure S7  For uterine cancers in B+, quasipoisson ecdos and dose models are displayed as in Figure S5.  Figure S8  For uterine cancers in A+, B+, C+, the preferred quasipoisson model Q5se (Table 2) is selected in each dose range. RR and 90% CIs are shown at age 60 and since 15 or 30. RR is plotted against since at age 60, and against age at since 20. Shadings are as in Figure S5. Points with dose ≤ 0.5 mGy are omitted.  Figure S9  For thyroid cancers in B+, quasipoisson ecdos and dose models are displayed as in Figure S5. Figure S10  For thyroid cancers in A+, B+, C+, the preferred quasipoisson model is selected in each dose range. RR and 90% CIs are shown at age 60 and 75 for A+ and B+, and at agex 10 and 40 for C+. RR is plotted against age and agex. Shadings are as in Figure S5. Points with dose ≤ 0.5 mGy are omitted.  back to topTables Tables The tabs within this Excel workbook are:  **Table S1** Cross-validation, all solid cancers in A+-  **Table S2** Preferred Poisson models, all solid cancers in A+-, B+-, C+-  **Table S3** Neutron RBE, Poisson models, all solid cancers in A+-, B+-, C+-  **Table S4** Excluding genital or respiratory cancers, Poisson models in A+-, B+-, C+-  **Table S5** Urban (proximal) data, Poisson models, all solid cancers in A, B, C  **Table S6** Hiroshima and Nagasaki, Poisson models, all solid cancers in A+-, B+-, C+-  **Table S7** Q2e and Q2d, quasipoisson models, 21 sites in A+-, B+-, C+-  **Table S8** Preferred quasipoisson models, 21 sites in A+-, B+-, C+-  **Table S9** Thyroid, comparisons with selected literature  Each tab contains explanatory notes  back to topCodes**To run a code file, first create a folder Documents/mini. Then right click on a link and save the file, under the same name and as a \*.txt file, to the folder.** **Code files contain comments and are described in the Appendices.**  code1.txt  R code for dose response, Bayesian posterior 90% CIs, and simulations as in Figure 2 (main text), centre panel (ML)  code2.txt  R code for cross-validation, as in Table S1  code3.txt  R code for comparison of ability to capture true dose response as in Figure 4 (main text)  code4.txt  R code for impact of lognormal dose errors as in Figure S4, left panel (P5se)  back to topData**These data files are required when running code. Right click on a link and save the file, under the same name and as a \*.txt file, to the Documents/mini folder.** soldat20x.txt  Data (M/F) for all solid cancers in A+. Codes using this data select for sex (and distcat).  soldat100x.txt  Data (M/F) for all solid cancers in B+. Codes using this data select for sex (and distcat).  back to topOutput**To use these output files when running a code, right click on a link and save the file, under the same name and as a \*.txt file, to the Documents/mini folder.** code1out.txt  output file for simulations in code1.txt  code2out1.txt  first output file for simulations in code2.txt  code2out2.txt  second output file for simulations in code2.txt  code3out1.txt  first output file for simulations in code3.txt  code3out2.txt  second output file for simulations in code3.txt  code4out.txt  output file for simulations in code4.txt |
