# Supplementary figures and images for: Low dose radiation risks for women surviving the a-bombs in Japan: generalized additive model
Source: Environ Health. 2016 Nov 24;15:112. doi: 10.1186/s12940-016-0191-3 (PMC5121957; doi:10.1186/s12940-016-0191-3)

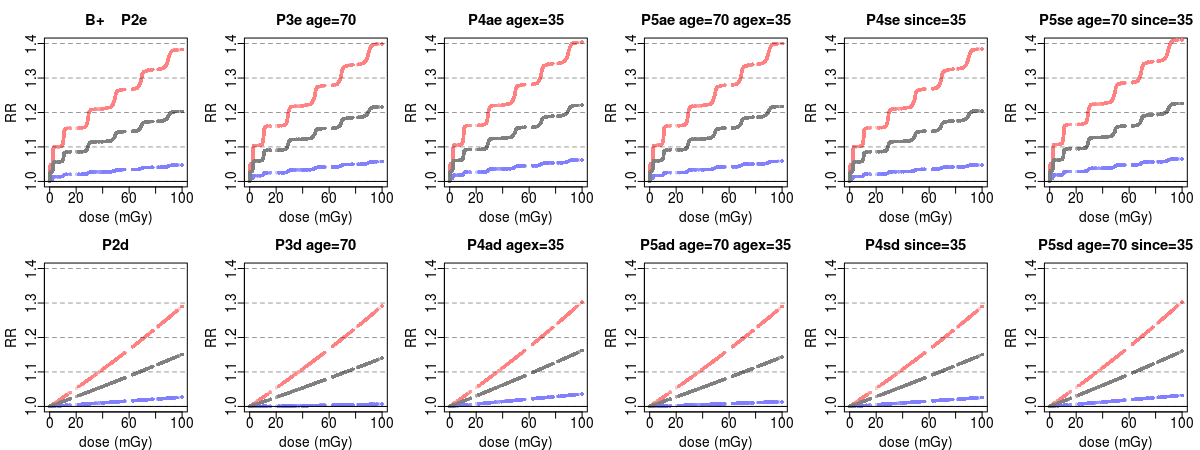

Supplement: Additional file 1 — Additional files are linked from a mini-website available with the online version of this paper. They consist of 1 PDF file with the Appendices, 10 images, 1 Excel workbook with 9 tables, 4 code files, 2 data files, and 6 output files. (ZIP 3727 kb) [file 12940_2016_191_MOESM1_ESM.zip › FigS1.tif]

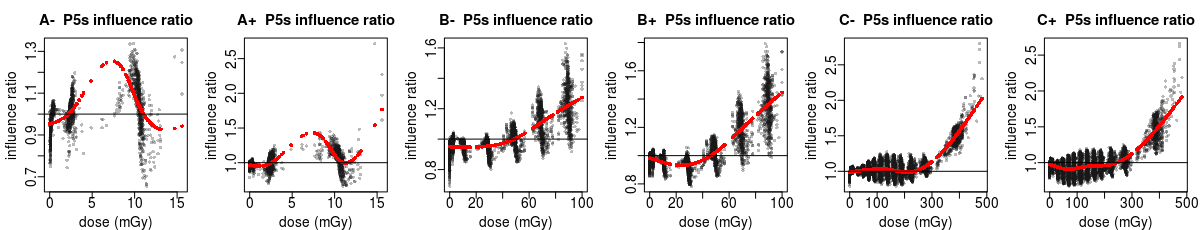

Supplement: Additional file 1 — Additional files are linked from a mini-website available with the online version of this paper. They consist of 1 PDF file with the Appendices, 10 images, 1 Excel workbook with 9 tables, 4 code files, 2 data files, and 6 output files. (ZIP 3727 kb) [file 12940_2016_191_MOESM1_ESM.zip › FigS2.tif]

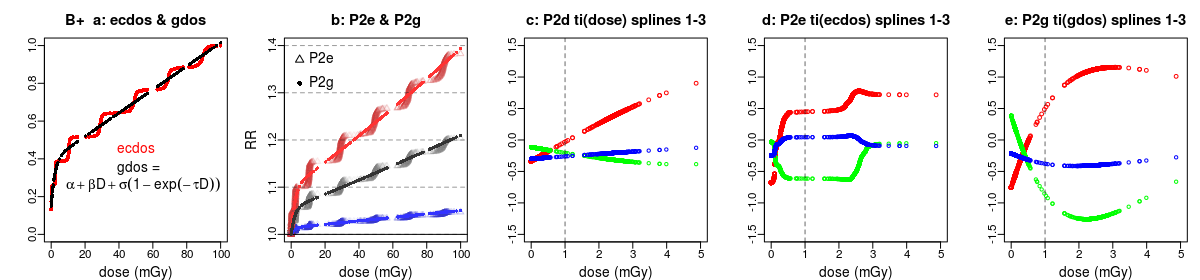

Supplement: Additional file 1 — Additional files are linked from a mini-website available with the online version of this paper. They consist of 1 PDF file with the Appendices, 10 images, 1 Excel workbook with 9 tables, 4 code files, 2 data files, and 6 output files. (ZIP 3727 kb) [file 12940_2016_191_MOESM1_ESM.zip › FigS3.tif]

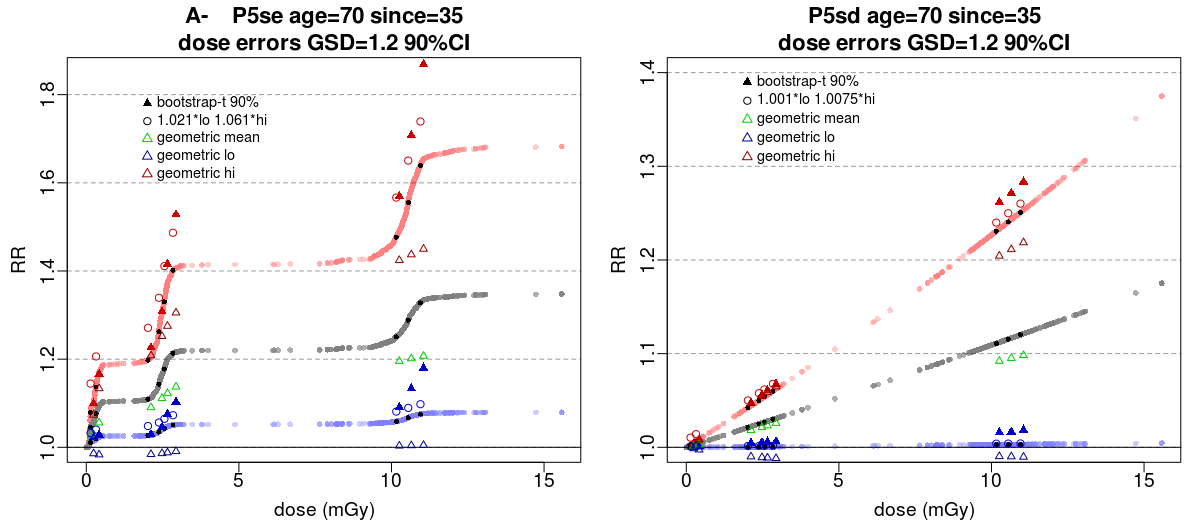

Supplement: Additional file 1 — Additional files are linked from a mini-website available with the online version of this paper. They consist of 1 PDF file with the Appendices, 10 images, 1 Excel workbook with 9 tables, 4 code files, 2 data files, and 6 output files. (ZIP 3727 kb) [file 12940_2016_191_MOESM1_ESM.zip › FigS4.tif]

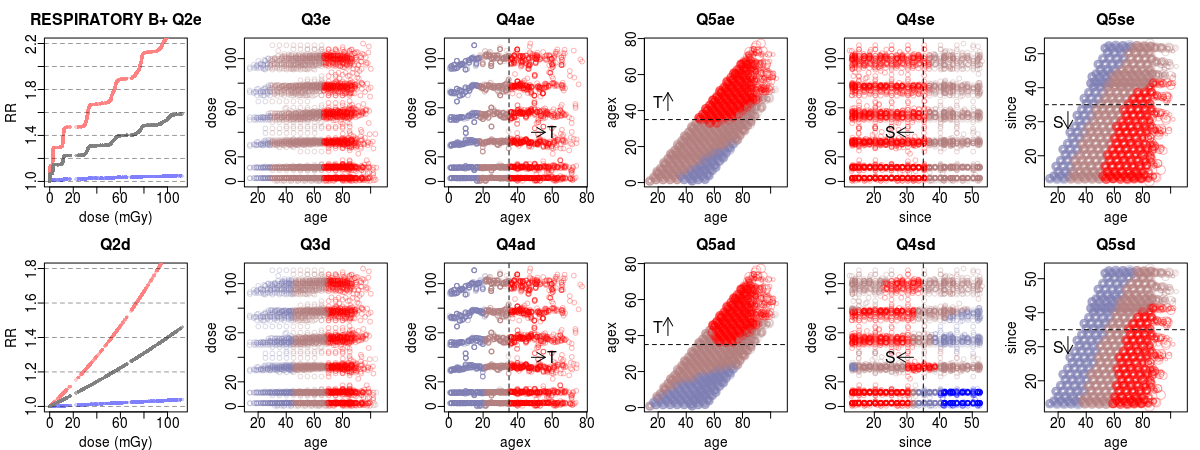

Supplement: Additional file 1 — Additional files are linked from a mini-website available with the online version of this paper. They consist of 1 PDF file with the Appendices, 10 images, 1 Excel workbook with 9 tables, 4 code files, 2 data files, and 6 output files. (ZIP 3727 kb) [file 12940_2016_191_MOESM1_ESM.zip › FigS5.tif]

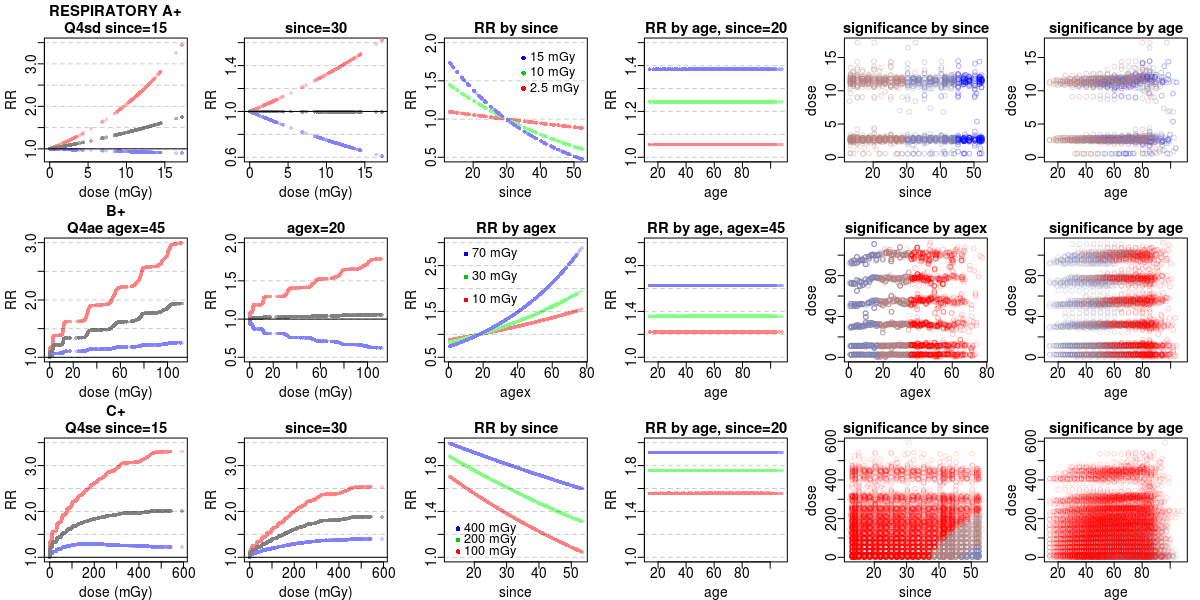

Supplement: Additional file 1 — Additional files are linked from a mini-website available with the online version of this paper. They consist of 1 PDF file with the Appendices, 10 images, 1 Excel workbook with 9 tables, 4 code files, 2 data files, and 6 output files. (ZIP 3727 kb) [file 12940_2016_191_MOESM1_ESM.zip › FigS6.tif]

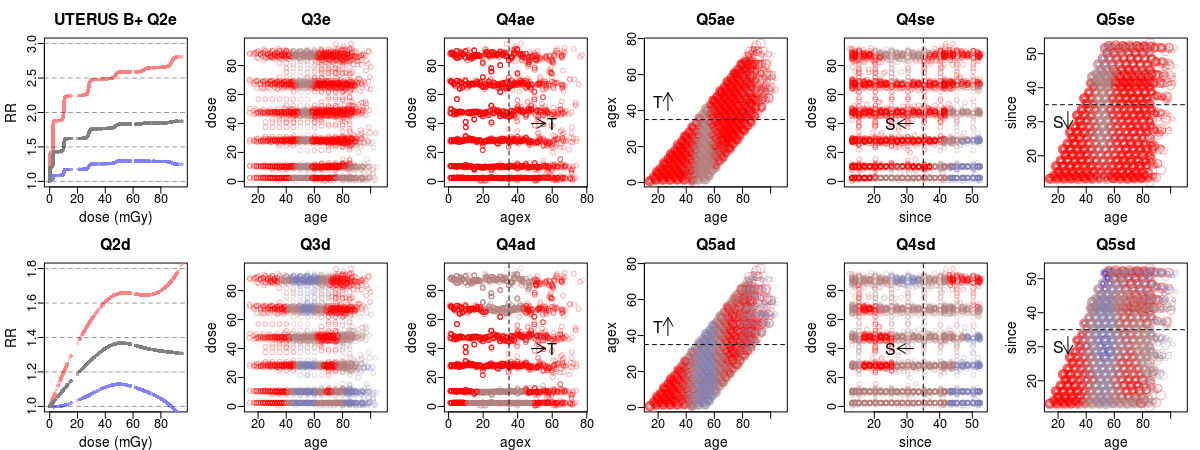

Supplement: Additional file 1 — Additional files are linked from a mini-website available with the online version of this paper. They consist of 1 PDF file with the Appendices, 10 images, 1 Excel workbook with 9 tables, 4 code files, 2 data files, and 6 output files. (ZIP 3727 kb) [file 12940_2016_191_MOESM1_ESM.zip › FigS7.tif]

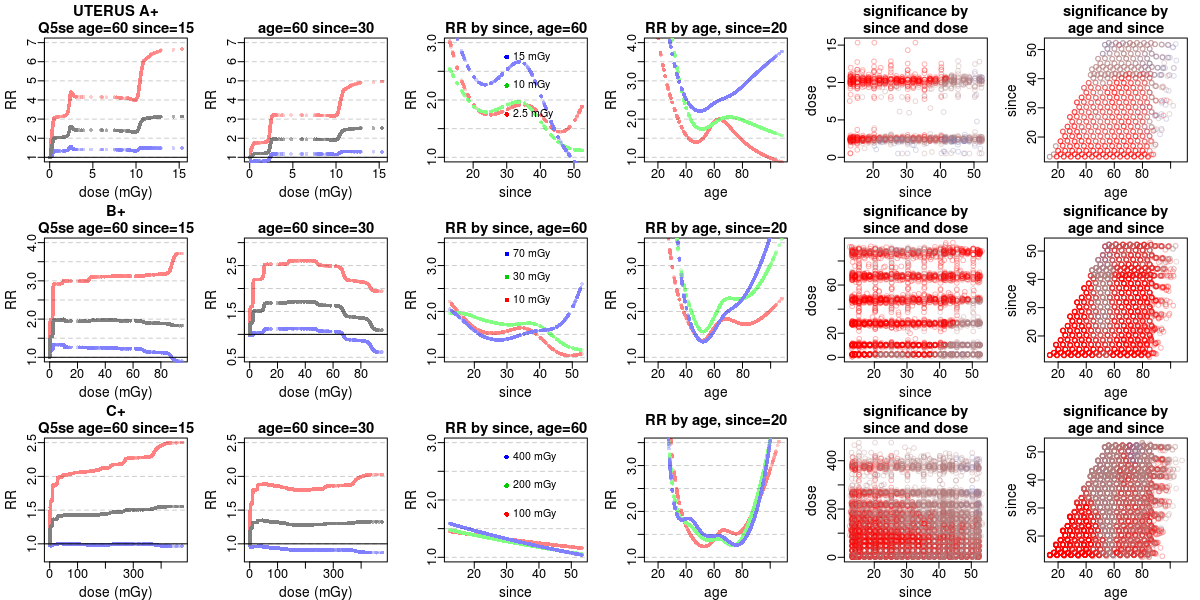

Supplement: Additional file 1 — Additional files are linked from a mini-website available with the online version of this paper. They consist of 1 PDF file with the Appendices, 10 images, 1 Excel workbook with 9 tables, 4 code files, 2 data files, and 6 output files. (ZIP 3727 kb) [file 12940_2016_191_MOESM1_ESM.zip › FigS8.tif]

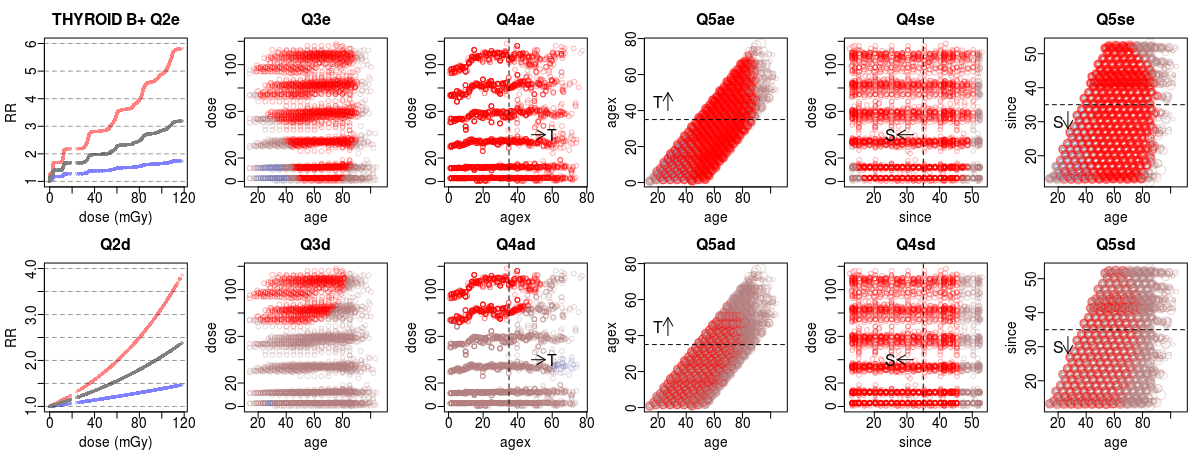

Supplement: Additional file 1 — Additional files are linked from a mini-website available with the online version of this paper. They consist of 1 PDF file with the Appendices, 10 images, 1 Excel workbook with 9 tables, 4 code files, 2 data files, and 6 output files. (ZIP 3727 kb) [file 12940_2016_191_MOESM1_ESM.zip › FigS9.tif]

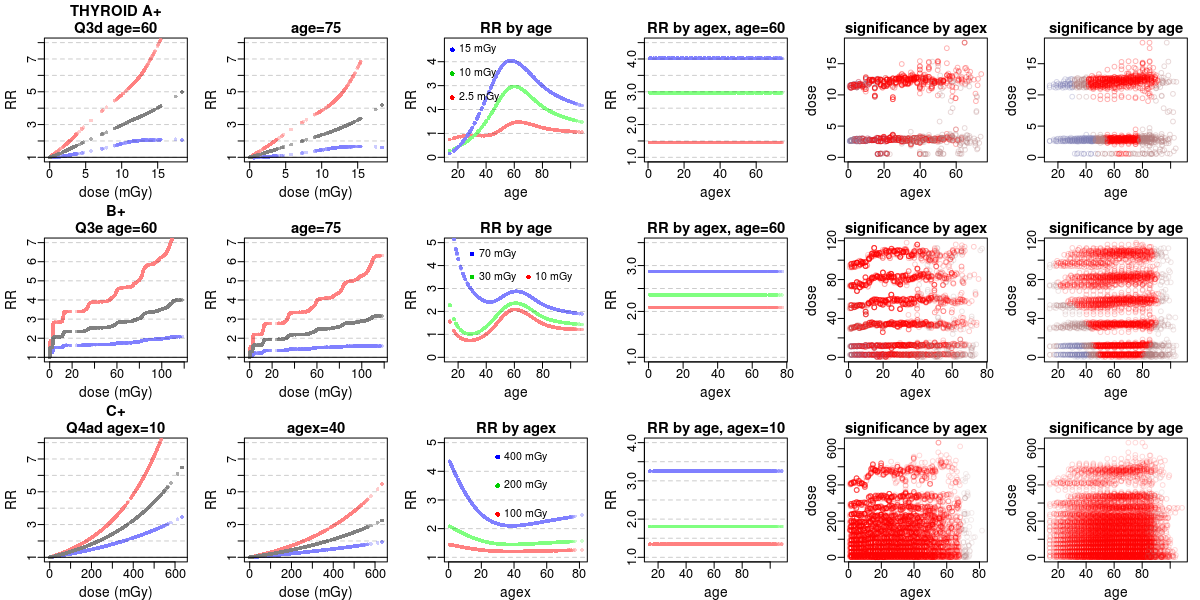

Supplement: Additional file 1 — Additional files are linked from a mini-website available with the online version of this paper. They consist of 1 PDF file with the Appendices, 10 images, 1 Excel workbook with 9 tables, 4 code files, 2 data files, and 6 output files. (ZIP 3727 kb) [file 12940_2016_191_MOESM1_ESM.zip › FigS10.tif]
